# Potential molecular links between triclosan exposure and osteoarthritis: An integrative systems toxicology study

**DOI:** 10.1016/j.isci.2026.117868

**Published:** 2026-10-13

**Authors:** Xijie Tang, Haijia Xu, Xiangzhong Liu, Yi Yang, Zhanghua Li

**Affiliations:** 1 Department of Orthopaedics, Wuhan Third Hospital, Wuhan 430000, China; 2 Hubei Key Laboratory of Exercise Training and Monitoring, Department of Sports Medicine, Wuhan Sports University, Wuhan 430000, China

**Keywords:** triclosan, osteoarthritis, network toxicology, machine learning, molecular docking, molecular dynamics simulation

## Abstract

Osteoarthritis (OA) is a chronic disease affecting the entire joint, influenced by a combination of inflammatory, metabolic, immune, and environmental factors. Triclosan (TCS), a commonly used antimicrobial compound, has been linked to oxidative and immune disturbances; however, its molecular relevance to OA remains to be elucidated. This study integrated network toxicology, bulk transcriptomics, cross-cohort machine learning prioritization, immune infiltration analysis, single-cell transcriptomic analysis, molecular docking, molecular dynamics simulations, and primary chondrocyte experiments. Thirty candidate genes related to both TCS and OA were identified, with CBR3, MAOA, MMP13, and AVPR1A being prioritized. Among these, CBR3 exhibited the most stable contribution to model predictions, widespread expression across OA chondrocyte subpopulations, and significant associations with inferred immune-cell composition. Supporting molecular docking and dynamics analyses indicated structural compatibility and dynamic feasibility between TCS and the four candidate proteins. In primary rat chondrocytes, low-dose TCS heightened reactive oxygen species (ROS) accumulation, upregulated Cbr3 and Mmp13, downregulated Col2a1, Maoa, and Avpr1a, and increased CBR3 protein levels, with specific redox-related and matrix-related alterations becoming more pronounced under IL-1β stimulation. These findings contribute to a model that links TCS-associated redox disturbances to OA-relevant chondrocyte stress and matrix remodeling, providing candidate molecular connections for future mechanistic validation.

## Highlights

• Systems toxicology revealed potential links between triclosan and osteoarthritis
• CBR3, MAOA, MMP13, and AVPR1A were prioritized as candidate genes
• Low-dose TCS increased ROS and altered OA markers in primary chondrocytes
• CBR3 showed the strongest computational and experimental support

## Graphical abstract

**Figure:**
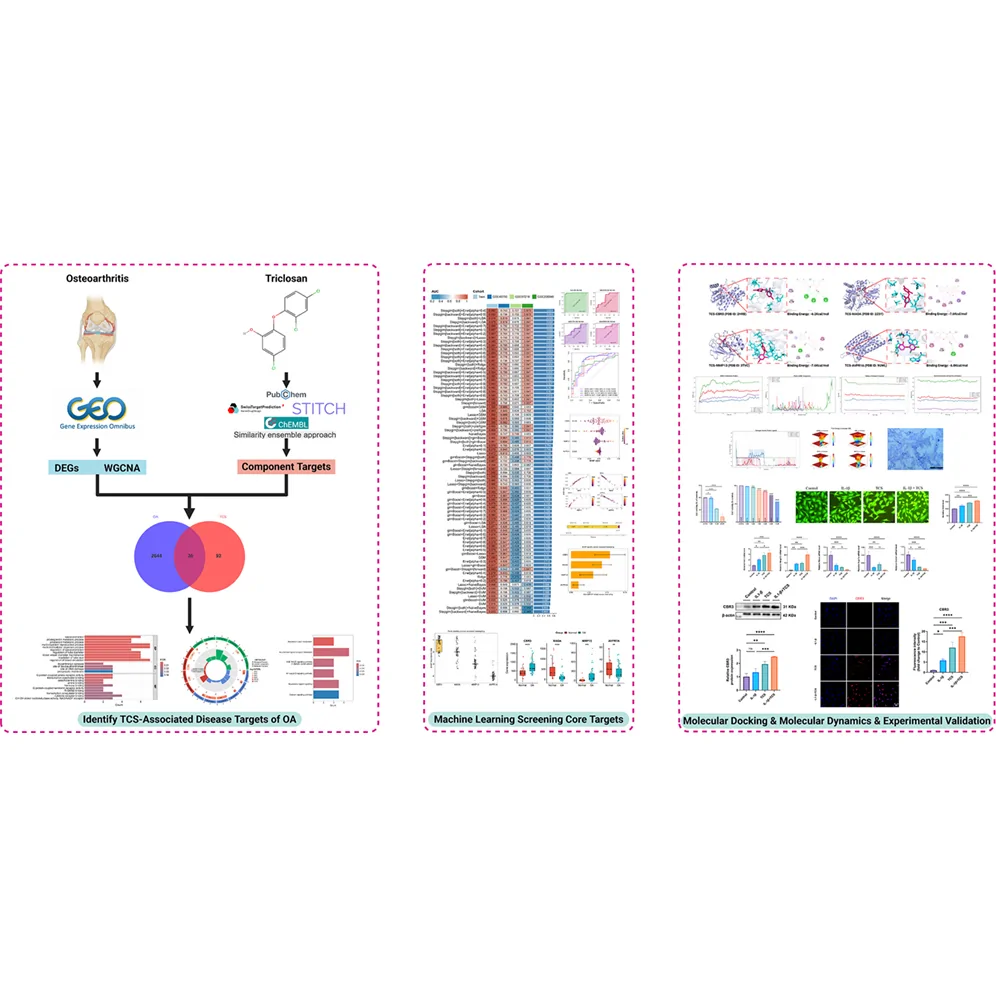

## Teaser

Molecular genetics; Cell biology; Transcriptomics

## Introduction

Osteoarthritis (OA) is increasingly recognized as a chronic whole-joint disease, encompassing more than just passive cartilage wear. OA affects the synovium, subchondral bone, and periarticular tissues, with inflammatory, immune, metabolic, vascular, and neuroendocrine processes playing roles in disease initiation and progression.^1^^,^^2^ These interconnected processes shape the microenvironment of the osteoarthritic joint, impacting both structural degeneration and clinical symptoms.^3^^,^^4^

Within this context, environmental exposures have emerged as potential modifiers of OA susceptibility and progression. Evidence from both population studies and experiments indicates that pollutants, such as phenols, phthalates, and ambient particulate matter, may affect OA risk through inflammatory, metabolic, vascular, and immune-related pathways.^5^^,^^6^ However, most reported exposure-disease relationships are primarily descriptive or association based, and the molecular mechanisms linking environmental chemicals, joint tissues, and immune-stromal interactions remain inadequately defined.

Triclosan (TCS), a synthetic broad-spectrum antimicrobial used in various consumer products, persists in the environment and is frequently detected in human biological samples, especially urine, indicating widespread exposure.^7^ Toxicological studies have linked TCS to endocrine disruption, oxidative stress, immune dysregulation, metabolic disturbances, and vascular dysfunction.^8^ Several of these processes overlap with known features of OA pathophysiology, yet the molecular pathways through which TCS-related disturbances may interact with the osteoarthritic joint microenvironment are not fully understood.

Clarifying this relationship necessitates approaches that capture system-level exposure biology rather than focusing on isolated pathways. Network toxicology and machine learning prioritization can integrate chemical-target information with disease-associated transcriptomic changes, while immune infiltration analysis and single-cell transcriptomics provide tissue- and cell-specific context.^9^^,^^10^^,^^11^ Molecular docking and dynamics simulations assess the predicted structural compatibility between TCS and prioritized candidate proteins. These methodologies form a framework for generating experimentally testable hypotheses regarding the molecular events associated with environmental chemical exposure.^12^

This study integrated network toxicology, bulk transcriptomic analysis, machine learning, single-cell transcriptomic analysis, immune infiltration profiling, molecular interaction modeling, and primary chondrocyte experiments to investigate the molecular associations between TCS exposure and OA. This workflow prioritized CBR3, MAOA, MMP13, and AVPR1A as candidate genes connecting predicted TCS-related targets with OA-associated transcriptomic alterations. Subsequent cell-based experiments examined the impact of low-dose TCS on reactive oxygen species (ROS) accumulation and OA-relevant molecular markers in primary chondrocytes. Rather than establishing a definitive causal pathway, this study offers a systems-level, hypothesis-generating framework for understanding how TCS-associated molecular perturbations may relate to redox, immune, and matrix-related processes relevant to OA.

## Results

### Identification of OA-related genes

To identify OA-associated transcriptomic alterations, GSE55235, GSE55457, and GSE82107 were integrated as the training cohort. Principal component analysis (PCA) showed that samples from the three datasets were clearly separated before batch correction, whereas samples from different datasets became more intermixed after ComBat correction, indicating reduced inter-dataset batch effects (Figure 1A).

**Figure 1:**
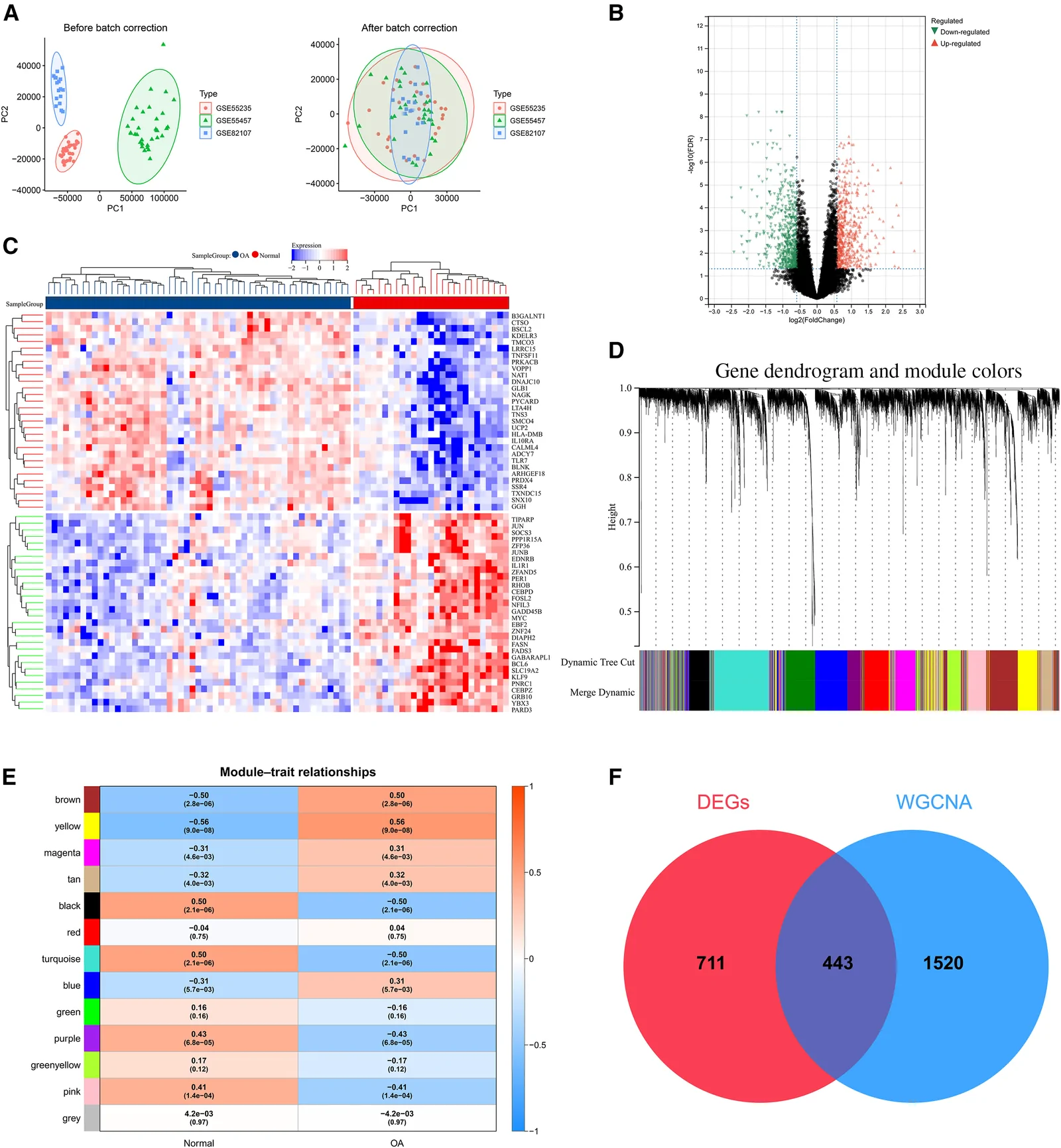
Identification of OA-related genes (A) PCA of the integrated GSE55235, GSE55457, and GSE82107 datasets before and after batch correction using the ComBat algorithm. (B) Volcano plot showing DEGs between OA and normal samples based on the criteria of FDR <0.05 and |log2FC| > 0.585. (C) Heatmap showing the expression patterns of representative DEGs between OA and normal samples. (D) Gene dendrogram and module colors generated by weighted gene co-expression network analysis (WGCNA). (E) Heatmap showing correlations between module eigengenes and OA and normal status. The brown, yellow, magenta, and tan modules were selected as OA-associated modules. (F) Venn diagram showing the union of DEGs and genes from selected OA-associated WGCNA modules to generate the OA-related gene set.

Differential expression analysis was performed using the batch-corrected training cohort. Using the criteria of false discovery rate (FDR) < 0.05 and |log2 fold change (log2FC)| > 0.585, a total of 1,154 differentially expressed genes (DEGs) were identified between OA and normal samples. The volcano plot showed the distribution of upregulated and downregulated genes, and the heatmap of representative DEGs revealed distinct expression patterns between OA and normal samples (Figures 1B and 1C).

Weighted gene co-expression network analysis (WGCNA) was further performed using the same batch-corrected expression matrix to identify OA-associated co-expression modules. Based on the scale-free topology criterion, β = 7 was selected for network construction. Dynamic tree cutting with a minimum module size of 100 identified 13 gene modules after module merging (Figure 1D). Module-trait correlation analysis showed that the brown, yellow, magenta, and tan modules were positively associated with OA status and showed inverse correlations with the normal phenotype; therefore, these modules were selected as OA-associated modules for subsequent analysis (Figure 1E).

Finally, genes from the DEG set and the selected OA-associated WGCNA modules were combined as a union to generate a comprehensive OA-related gene set. In total, 2,674 OA-related genes were retained for downstream integration with predicted TCS-related targets (Figure 1F).

### Identification of predicted TCS-related target genes

The chemical structure of TCS was obtained from PubChem and used for subsequent target prediction (Figure 2A). Putative TCS-related target genes were collected from SwissTargetPrediction, STITCH, ChEMBL, and the Similarity Ensemble Approach (SEA) database. After integrating the target lists from these sources and removing duplicate entries, 122 non-redundant predicted TCS-related target genes were obtained (Figure 2B).

**Figure 2:**
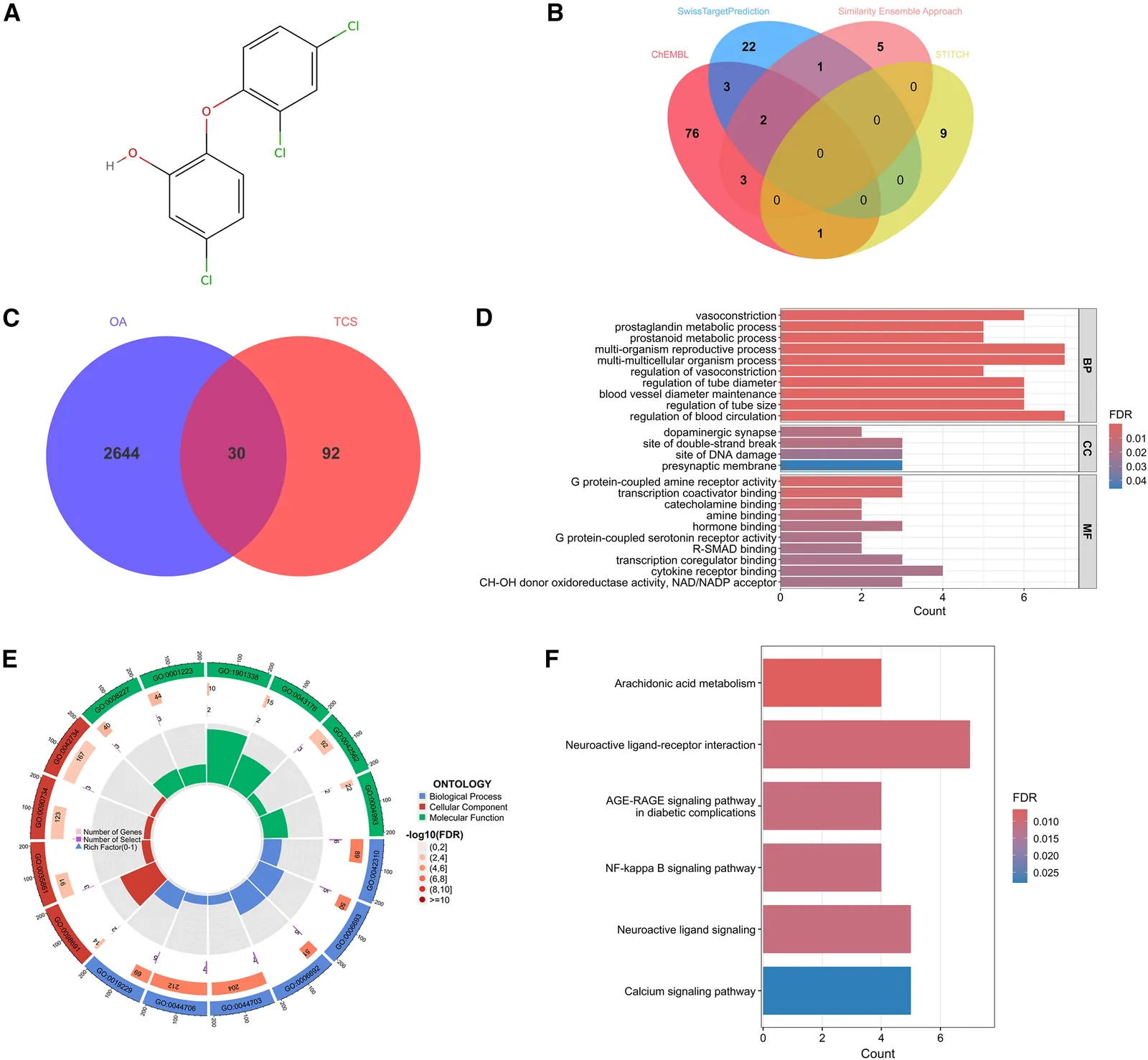
Identification and enrichment analysis of shared TCS-OA candidate genes (A) Chemical structure of TCS. (B) Venn diagram showing predicted TCS-related target genes obtained from ChEMBL, SwissTargetPrediction, Similarity Ensemble Approach (SEA), and STITCH. (C) Venn diagram showing the intersection between predicted TCS-related target genes and OA-related genes, resulting in 30 shared TCS- and OA-related candidate genes. (D) GO enrichment analysis of the shared TCS- and OA-related candidate genes, including biological process (BP), cellular component (CC), and molecular function (MF) categories. (E) Circular GO enrichment plot summarizing representative enriched GO terms and their significance. (F) KEGG pathway enrichment analysis of the shared TCS- and OA-related candidate genes.

### Shared TCS-OA candidate genes and enrichment analysis

To identify candidate genes shared between predicted TCS-related targets and OA-associated transcriptomic alterations, the predicted TCS-related target gene set was intersected with the OA-related gene set identified earlier. This analysis identified 30 shared TCS- and OA-related candidate genes, which were used for subsequent enrichment analysis and machine-learning-based prioritization (Figure 2C).

Functional enrichment analysis was then performed to characterize the biological context of these 30 shared candidate genes. Gene Ontology (GO) enrichment analysis showed that these genes were mainly associated with prostaglandin/prostanoid metabolism, vascular-regulatory processes, neuroactive receptor-related molecular functions, cytokine-related binding, transcriptional regulation, and oxidoreductase activity. Enriched cellular component terms also involved synapse-related and DNA damage-associated compartments (Figures 2D and 2E).

Kyoto Encyclopedia of Genes and Genomes (KEGG) enrichment analysis further highlighted several OA-relevant biological pathways, including arachidonic acid metabolism, NF-κB signaling, AGE-RAGE signaling, calcium signaling, and neuroactive ligand-receptor interaction (Figure 2F). Overall, these results suggested that the shared TCS- and OA-related candidate genes were functionally associated with lipid inflammatory mediator metabolism, inflammatory signaling, redox-related molecular functions, calcium-dependent signaling, and neuroactive ligand-related pathways.

### Machine learning prioritization and Shapley additive explanations interpretation

To prioritize robust candidate genes among the 30 shared TCS- and OA-related candidate genes, 113 predefined machine learning pipelines were constructed and internally assessed using repeated stratified 5-fold cross-validation in the training cohort. Each pipeline was subsequently fitted using the full training cohort and evaluated in three independent external cohorts. For final pipeline prioritization, the mean area under the receiver operating characteristic curve (AUC) across the training cohort and the three external evaluation cohorts was used as the summary cross-cohort performance metric. Among these pipelines, the Stepglm[both] + Enet[alpha = 0.4] model showed the highest mean AUC and was therefore selected as the final predictive model (Figure 3A).

**Figure 3:**
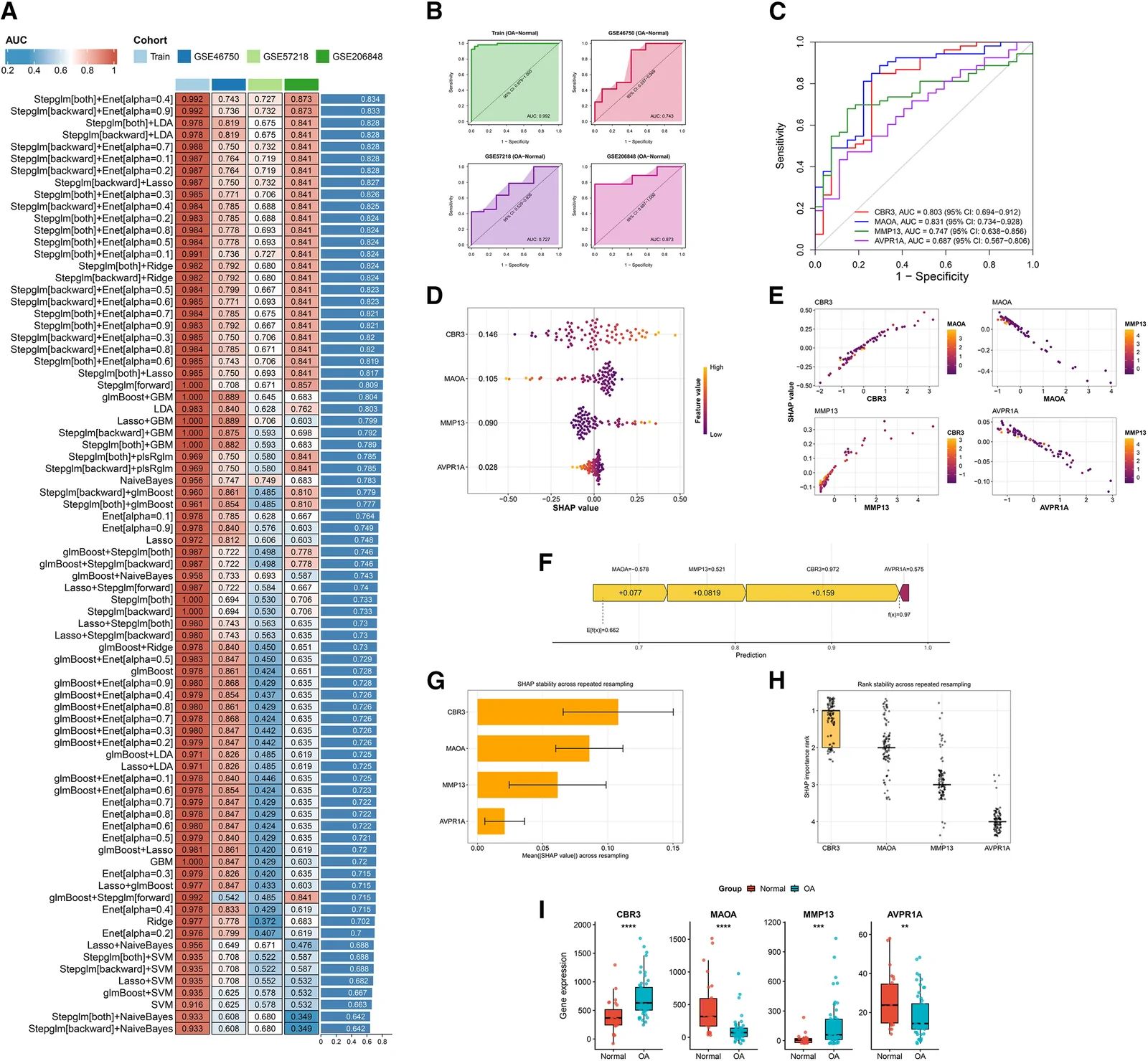
Machine learning prioritization and SHAP interpretation of shared TCS- and OA-related candidate genes (A) Heatmap summarizing the AUC performance of 113 predefined machine learning pipelines across the training cohort and three independent external evaluation cohorts. (B) ROC curves showing the predictive performance of the final selected machine learning model in the training cohort and three independent external evaluation cohorts. (C) ROC curves showing the discriminative performance of individual prioritized candidate genes, including CBR3, MAOA, MMP13, and AVPR1A. (D) SHAP summary plot showing the contribution of the four prioritized candidate genes to model prediction. (E) SHAP dependence plots showing gene-specific relationships between expression levels and SHAP values. (F) Representative SHAP force plot illustrating the combined contribution of the prioritized candidate genes to an individual model prediction. (G) Mean absolute SHAP values of the prioritized candidate genes across repeated stratified resampling analyses. (H) Rank-stability analysis of gene importance across repeated resampling iterations. (I) Boxplots comparing the expression levels of CBR3, MAOA, MMP13, and AVPR1A between normal and OA samples. Statistical significance in (I) was assessed using the Wilcoxon rank-sum test. ns, not significant; ∗*p* < 0.05; ∗∗*p* < 0.01; ∗∗∗*p* < 0.001; ∗∗∗∗*p* < 0.0001.

The selected model showed high discriminative performance in the training cohort, with an AUC of 0.992. In the three external evaluation cohorts, the model achieved AUC values of 0.743 in GSE46750, 0.727 in GSE57218, and 0.873 in GSE206848, supporting acceptable cross-cohort predictive performance. The corresponding 95% confidence intervals were displayed in the receiver operating characteristic (ROC) plots (Figure 3B). Based on the genes retained by this model, CBR3, MAOA, MMP13, and AVPR1A were identified as four prioritized candidate genes. ROC analysis of the individual candidate genes showed moderate discriminative ability, with AUC values of 0.803 for CBR3, 0.831 for MAOA, 0.747 for MMP13, and 0.687 for AVPR1A. The 95% confidence intervals for the individual-gene ROC curves were also shown in the plot (Figure 3C). These results suggested that although individual genes showed varying predictive performance, joint modeling of the four genes provided improved classification performance across cohorts.

Shapley additive explanations (SHAP) analysis was then performed to interpret the contribution of the four prioritized candidate genes to model prediction. The SHAP summary plot showed that CBR3 had the largest contribution to model output, followed by MAOA and MMP13, whereas AVPR1A showed a smaller but retained contribution (Figure 3D). SHAP dependence plots further illustrated gene-specific relationships between expression levels and model contribution, supporting heterogeneous predictive patterns among the four genes (Figure 3E). A representative SHAP force plot demonstrated the combined contribution of these genes to an individual prediction (Figure 3F).

To further assess the robustness of gene importance, SHAP analysis was repeated across 100 stratified resampling iterations. CBR3 showed the highest and most stable mean absolute SHAP value, followed by MAOA and MMP13, whereas AVPR1A showed a relatively weaker contribution (Figure 3G). Rank-stability analysis similarly supported CBR3 and MAOA as consistently high-ranking predictors, with MMP13 showing intermediate stability and AVPR1A ranking lower across repeated resampling (Figure 3H).

Finally, the expression patterns of the four prioritized candidate genes were compared between OA and normal samples. CBR3 and MMP13 were significantly upregulated in OA samples, whereas MAOA and AVPR1A were significantly downregulated (Figure 3I). Together, these findings supported the prioritization of CBR3, MAOA, MMP13, and AVPR1A as candidate genes associated with OA-related transcriptomic differences within the shared TCS- and OA-related gene set.

### Single-cell expression patterns of prioritized candidate genes

To characterize the cellular distribution of the four prioritized candidate genes, OA-related single-cell RNA sequencing (RNA-seq) data were analyzed after quality control, normalization, dimensionality reduction, and batch correction. Uniform manifold approximation and projection (UMAP) visualization identified 20 cell clusters, which were further annotated into seven OA-related chondrocyte subpopulations, including fibrocartilage chondrocytes (FCs), homeostatic chondrocytes (HomCs), pre-hypertrophic chondrocytes (pre-HTCs), hypertrophic chondrocytes (HTCs), proliferative chondrocytes (ProCs), effector/inflammatory chondrocytes (ECs), and regulatory chondrocytes (RegCs) (Figure 4A).

**Figure 4:**
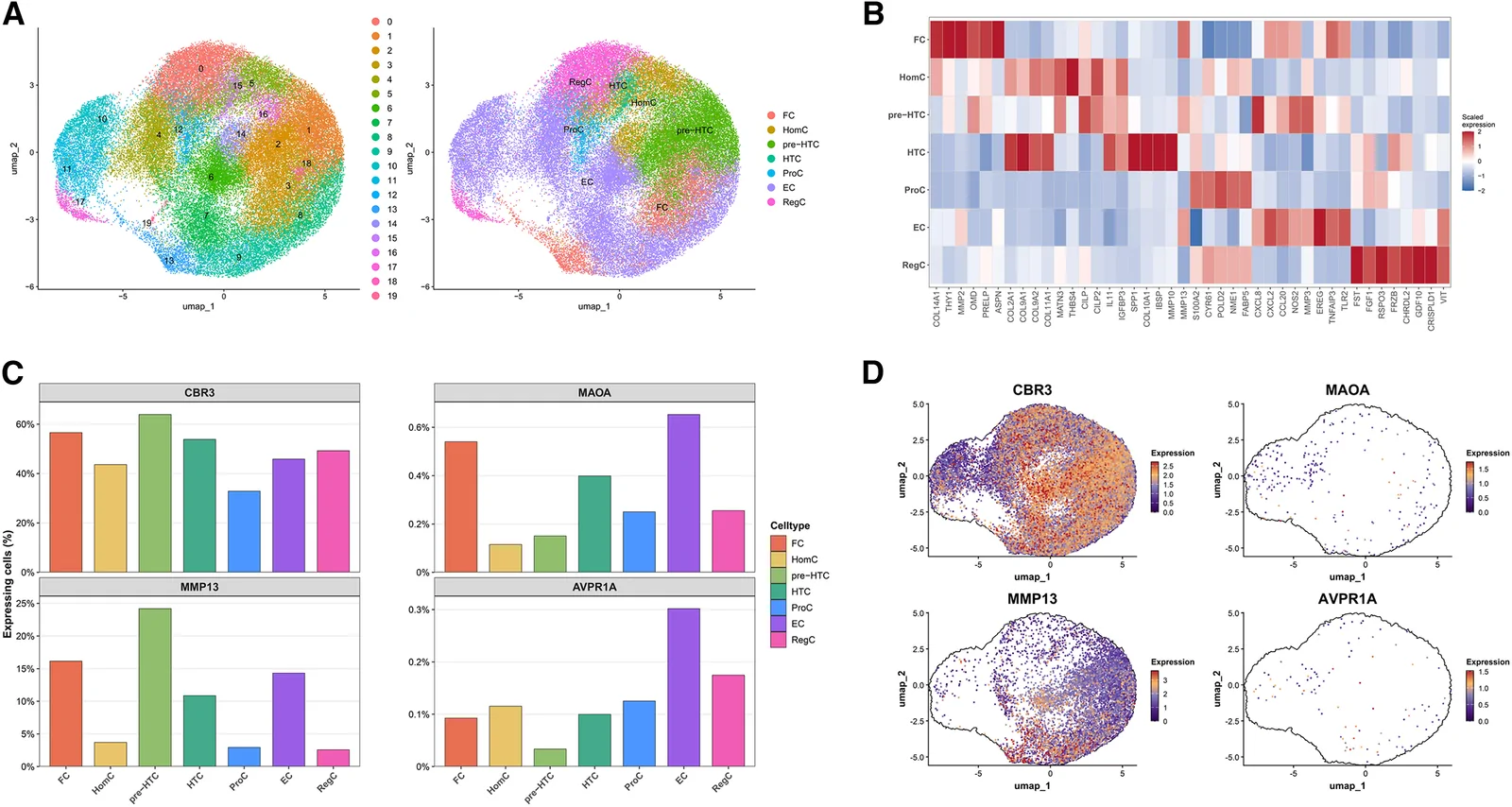
Single-cell expression patterns of prioritized candidate genes in OA chondrocyte subpopulations (A) UMAP visualization of single-cell RNA-seq data showing 20 unsupervised clusters and their annotation into seven OA-related chondrocyte subpopulations, including fibrocartilage chondrocytes (FCs), homeostatic chondrocytes (HomCs), pre-hypertrophic chondrocytes (pre-HTCs), hypertrophic chondrocytes (HTCs), proliferative chondrocytes (ProCs), effector/inflammatory chondrocytes (ECs), and regulatory chondrocytes (RegCs). (B) Heatmap showing representative marker gene expression patterns across the annotated chondrocyte subpopulations. (C) Bar plots showing the proportion of cells with detectable expression of CBR3, MAOA, MMP13, and AVPR1A across annotated chondrocyte subpopulations. (D) UMAP feature plots showing the expression distributions of CBR3, MAOA, MMP13, and AVPR1A across single cells.

The cell-subpopulation annotation was supported by representative marker gene expression patterns. The marker heatmap showed distinct enrichment of cluster-specific markers across the annotated chondrocyte subpopulations, supporting the annotation of FC, HomC, pre-HTC, HTC, ProC, EC, and RegC populations (Figure 4B).

The proportion of cells with detectable expression of the four prioritized candidate genes was then calculated across the annotated subpopulations. CBR3 showed broad expression across multiple chondrocyte subpopulations, with relatively high proportions of expressing cells in pre-HTCs, FCs, HTCs, RegCs, ECs, and HomCs. MMP13 showed more restricted but notable expression, particularly in pre-HTC, FC, EC, and HTC populations. In contrast, MAOA and AVPR1A were detected in only a small fraction of cells across all annotated subpopulations, indicating low-abundance and sparse expression at the single-cell level (Figure 4C).

UMAP-based feature plots were consistent with these expression patterns. CBR3 was broadly distributed across the UMAP space, whereas MMP13 showed a more localized expression pattern within specific chondrocyte regions. MAOA and AVPR1A displayed sparse and low-level expression signals, consistent with the low proportions of cells with detectable expression (Figure 4D). Together, these single-cell results indicated that the prioritized candidate genes exhibited distinct subpopulation-specific expression patterns in OA chondrocytes, with CBR3 showing the broadest distribution and MMP13 showing more selective enrichment in OA-associated chondrocyte subpopulations.

### Immune infiltration and gene-immune cell correlation analysis

To characterize immune-cell composition in OA and normal samples, CIBERSORT was used to estimate the relative abundance of 22 immune cell subtypes in the training cohort. The stacked bar plot showed substantial inter-sample heterogeneity in immune-cell composition, with macrophage subsets, mast cells, T cell subsets, plasma cells, and other immune populations contributing variably across samples (Figure 5A).

**Figure 5:**
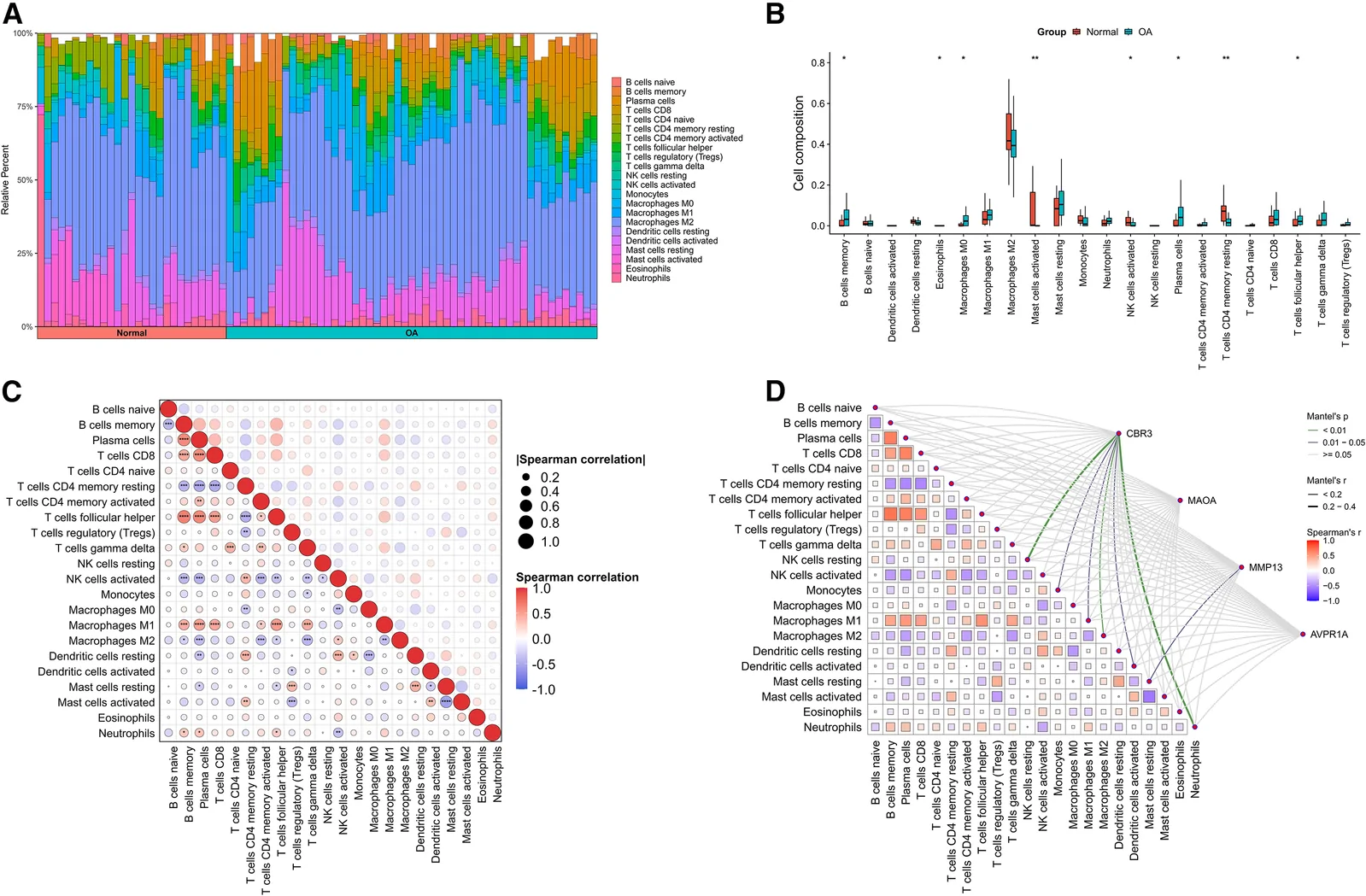
Immune infiltration patterns and gene-immune cell associations in OA (A) Stacked bar plot showing the relative abundance of 22 immune cell subtypes inferred by CIBERSORT across normal and OA samples. (B) Boxplots comparing the estimated abundance of immune cell subtypes between normal and OA samples. Statistical significance was assessed using Wilcoxon rank-sum tests followed by FDR correction. (C) Spearman correlation matrix showing pairwise correlations among estimated immune cell subtypes. (D) Combined gene-immune cell association plot showing relationships between the prioritized candidate genes and immune-cell composition. ns, not significant; ∗*p* < 0.05; ∗∗*p* < 0.01; ∗∗∗*p* < 0.001; ∗∗∗∗*p* < 0.0001.

Comparative analysis between OA and normal samples revealed significant differences in several immune cell subtypes after FDR correction. These altered populations included memory B cells, eosinophils, macrophage subsets, mast cell subsets, natural killer (NK) cells, plasma cells, CD4 memory T cell subsets, and T follicular helper cells, suggesting that OA samples exhibited an altered immune-cell composition compared with normal controls (Figure 5B).

Spearman correlation analysis was then performed to evaluate the relationships among estimated immune cell populations. The immune-cell correlation matrix showed coordinated positive and negative associations among multiple lymphoid and myeloid cell subsets, indicating structured covariation within the inferred immune microenvironment (Figure 5C).

The relationships between the four prioritized candidate genes and immune-cell composition were further evaluated using gene-immune cell correlation and Mantel-based association analysis. CBR3 showed the most extensive association pattern with multiple immune cell populations, whereas MAOA, MMP13, and AVPR1A displayed more limited and cell-subtype-specific association patterns (Figure 5D). These findings suggested that the prioritized candidate genes, particularly CBR3, were associated with OA-related immune-cell composition changes, although these correlations should be interpreted as associative rather than causal.

### Molecular docking analysis of TCS with prioritized candidate proteins

Molecular docking analysis was performed to evaluate the predicted binding interactions between TCS and the four prioritized candidate proteins, including CBR3, MAOA, MMP13, and AVPR1A. Experimentally resolved protein structures of CBR3, MAOA, MMP13, and AVPR1A were obtained from the Protein Data Bank (PDB), and TCS was docked into the putative ligand-binding regions of each protein.

TCS was predicted to form energetically favorable binding poses with all four candidate proteins, with predicted binding energies ranging from −6.2 to −7.6 kcal/mol. Specifically, the predicted binding energies were −6.2 kcal/mol for the TCS-CBR3 complex, −7.6 kcal/mol for the TCS-MAOA complex, −7.6 kcal/mol for the TCS-MMP13 complex, and −6.8 kcal/mol for the TCS-AVPR1A complex (Figures 6A–6D).

**Figure 6:**
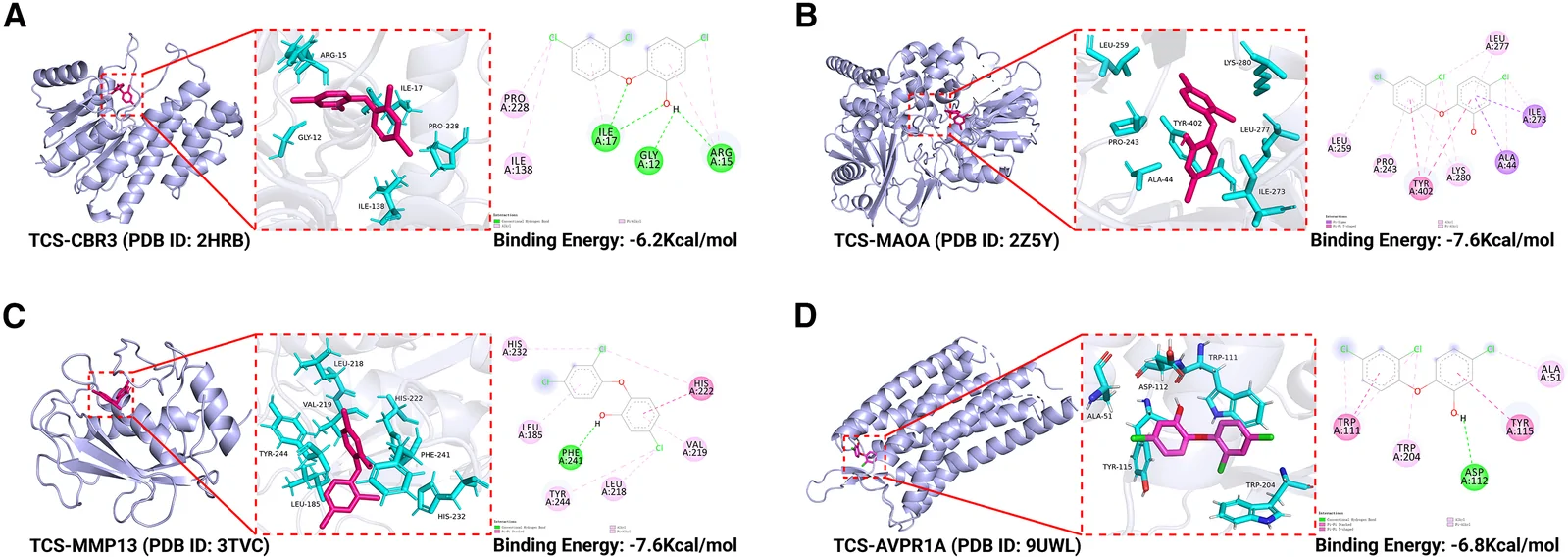
Molecular docking analysis of TCS with prioritized candidate proteins (A–D) Predicted docking conformations of TCS with CBR3, MAOA, MMP13, and AVPR1A. Each image shows the global docking pose, magnified binding-region view, and corresponding two-dimensional interaction diagram.

Visualization of the docking conformations showed that TCS could be accommodated within the predicted binding pockets of these proteins through multiple noncovalent interactions, including hydrophobic contacts and potential hydrogen-bond interactions. Among the four proteins, MAOA and MMP13 showed the lowest predicted binding energies, whereas CBR3 and AVPR1A also exhibited favorable docking scores under the same docking settings.

Together, these docking results suggested that TCS showed structural compatibility with the predicted binding regions of CBR3, MAOA, MMP13, and AVPR1A. These findings provide computational support for considering these proteins as putative TCS-interacting candidates, although the predicted interactions require further experimental validation.

### Molecular dynamics simulation and free energy landscape analysis

To assess the dynamic behavior of the predicted TCS-protein complexes, 100 ns molecular dynamics (MD) simulations were performed for TCS-CBR3, TCS-MAOA, TCS-MMP13, and TCS-AVPR1A. The resulting trajectories were analyzed to compare conformational stability and interaction dynamics among the four complexes. Protein root-mean-square deviation (RMSD) analysis showed that all four systems underwent an initial adjustment phase followed by relatively stable trajectories during the simulation. The TCS-CBR3 and TCS-MMP13 complexes maintained comparatively lower RMSD values, suggesting relatively stable protein backbone conformations. The TCS-AVPR1A complex showed moderate RMSD fluctuations, whereas the TCS-MAOA complex exhibited higher RMSD values, indicating greater conformational mobility under the simulated conditions (Figure 7A).

**Figure 7:**
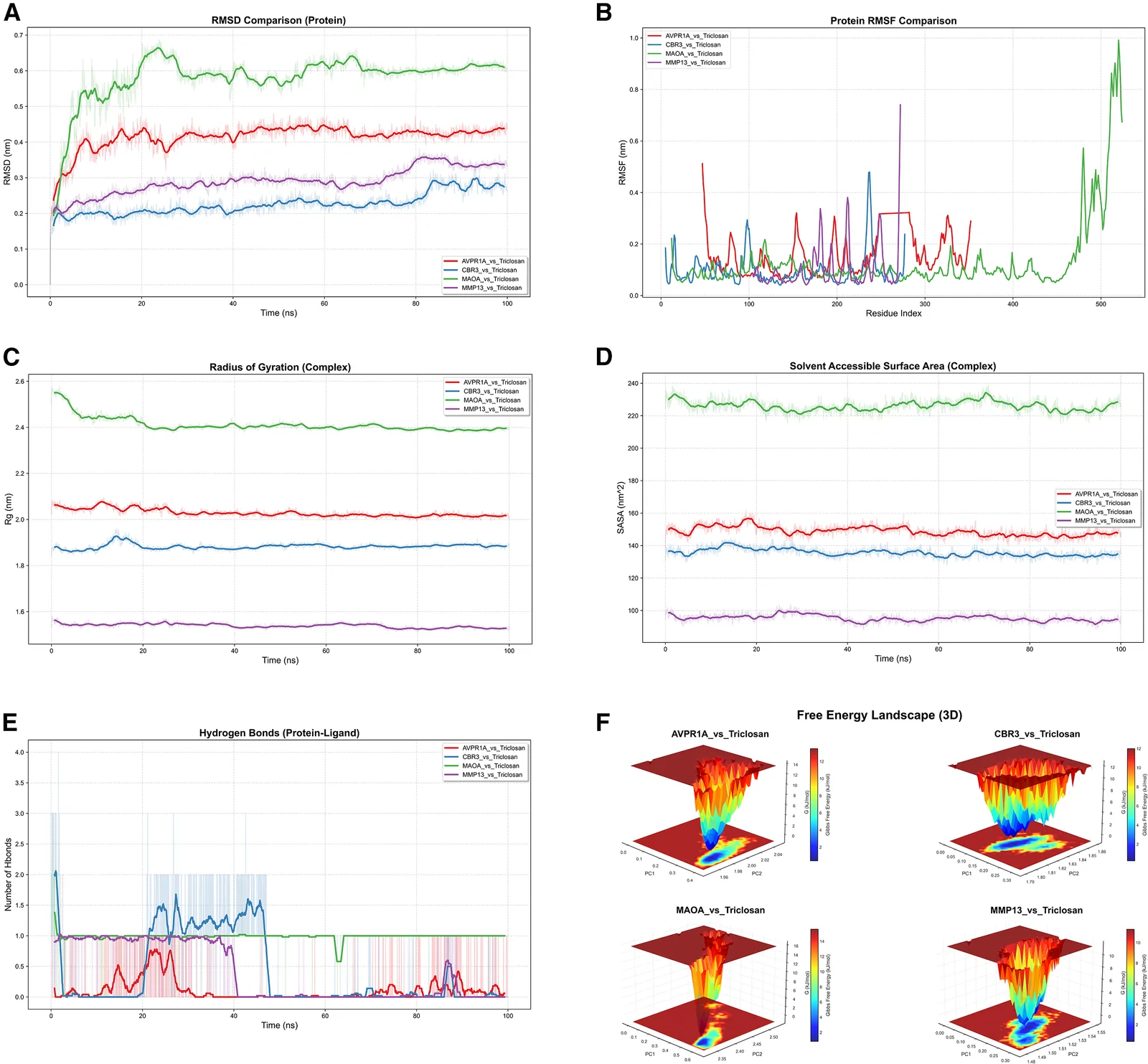
MD simulation and free energy landscape analysis of TCS-protein complexes (A) Protein RMSD trajectories of the TCS-CBR3, TCS-MAOA, TCS-MMP13, and TCS-AVPR1A complexes during molecular dynamics simulations. (B) Residue-level RMSF profiles of the four TCS-protein complexes. (C) Rg profiles showing the compactness of the complexes over time. (D) SASA trajectories of the complexes during the simulations. (E) Time-dependent hydrogen bond analysis between TCS and the candidate proteins. (F) FEL analysis of the four TCS-protein complexes.

Residue-level root-mean-square fluctuation (RMSF) analysis further showed that most residues in the four complexes displayed limited fluctuations, whereas higher flexibility was mainly observed in specific loop or terminal regions. This pattern indicated that local residue mobility varied among the complexes, while the overall protein structures remained generally stable during the simulations (Figure 7B). The radius of gyration (Rg) remained relatively stable for all four complexes throughout the simulations, suggesting that no obvious global unfolding or large-scale structural expansion occurred during the 100 ns trajectories (Figure 7C). Similarly, solvent-accessible surface area (SASA) values fluctuated within relatively narrow ranges for each system, further supporting the maintenance of overall structural compactness and solvent-exposure stability (Figure 7D).

Protein-ligand hydrogen bond analysis revealed dynamically fluctuating hydrogen-bond interactions between TCS and the candidate proteins. Intermittent hydrogen bonds were observed across the simulation trajectories, indicating that TCS-protein interactions were maintained in a dynamic rather than static manner (Figure 7E). Free energy landscape (FEL) analysis showed that each TCS-protein complex sampled defined low-energy conformational regions during the simulation. These low-energy basins suggested that the docked complexes could access energetically favorable conformational states under the simulated conditions (Figure 7F). Together, these MD results supported the conformational stability and dynamic feasibility of the predicted TCS interactions with CBR3, MAOA, MMP13, and AVPR1A, while further experimental validation remains necessary to confirm direct binding.

### Cellular responses to TCS in primary rat chondrocytes

Primary rat chondrocytes were first identified by toluidine blue staining, which showed glycosaminoglycan-rich matrix staining consistent with the chondrocytic phenotype (Figure 8A). Cell Counting Kit-8 (CCK-8) assays were then used to define a low-dose working range for TCS exposure. In the broad screening assay, 5 μM TCS had little effect on cell viability, whereas 10 and 20 μM TCS markedly reduced viability (Figure 8B). A refined dose screen further showed a gradual decline in viability from 5 to 10 μM TCS. Because 7 μM TCS retained approximately 88% cell viability while still representing a defined low-dose exposure condition, this concentration was selected for subsequent experiments (Figure 8C).

**Figure 8:**
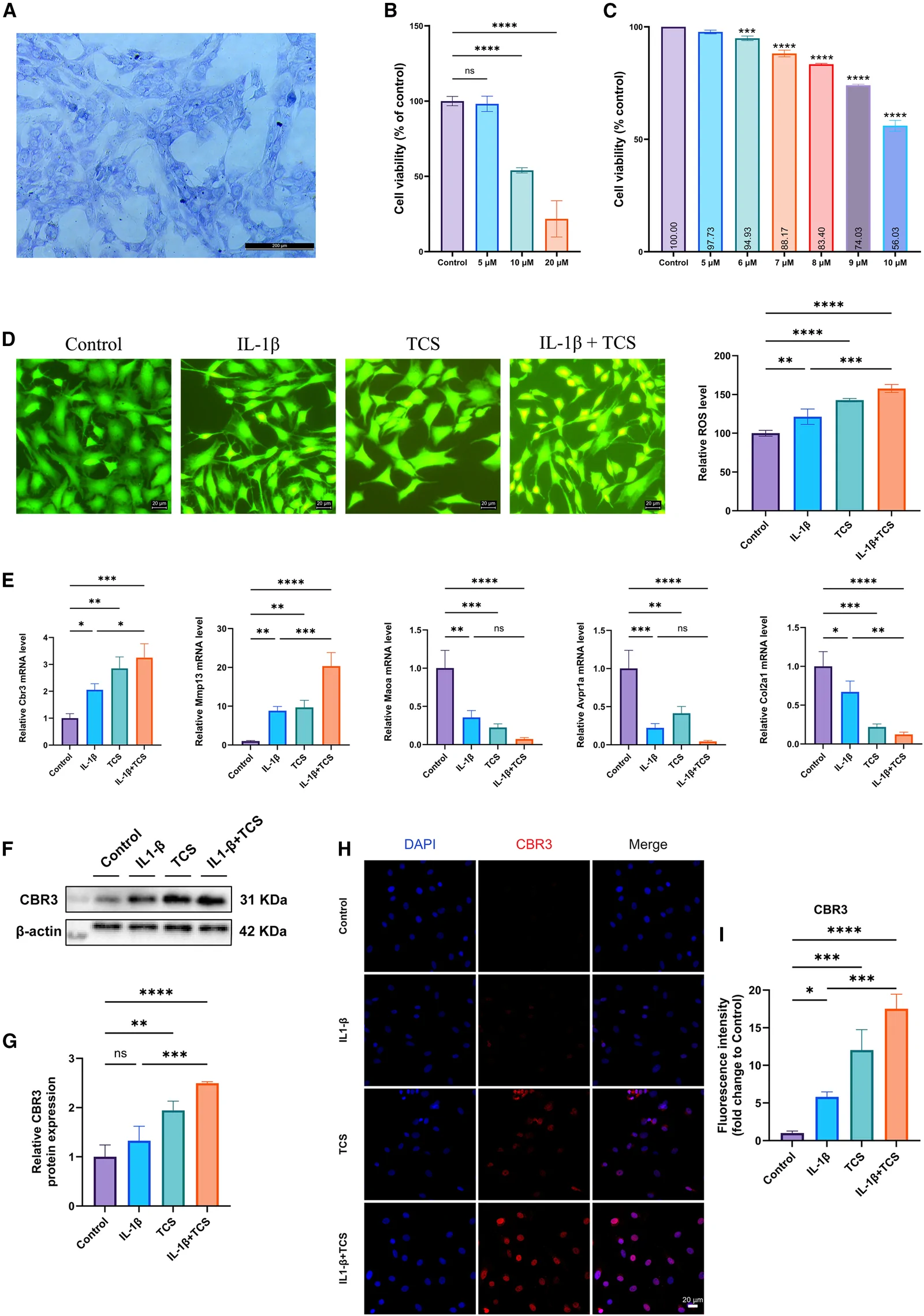
Cellular responses to TCS in primary rat chondrocytes (A) Representative toluidine blue staining image of primary rat chondrocytes. Positive staining indicated the presence of glycosaminoglycan-rich extracellular matrix. (B) Initial CCK-8 screening of primary rat chondrocyte viability following treatment with 0, 5, 10, or 20 μM TCS for 30 h. (C) Refined CCK-8 screening of cell viability following treatment with 0, 5, 6, 7, 8, 9, or 10 μM TCS for 30 h. (D) Representative DCFH-DA fluorescence images and quantitative analysis of intracellular ROS levels in the control, IL-1β, TCS, and IL-1β + TCS groups. (E) Real-time qPCR analysis of the relative mRNA expression levels of *Cbr3*, *Mmp13*, *Maoa*, *Avpr1a*, and *Col2a1* in primary rat chondrocytes following the indicated treatments. (F and G) Representative western blot bands of CBR3 and β-actin and densitometric quantification of relative CBR3 protein expression normalized to β-actin and expressed relative to control. (H and I) Representative immunofluorescence images of CBR3 expression and quantitative analysis of CBR3 fluorescence intensity in primary rat chondrocytes. Nuclei were counterstained with 4′,6-diamidino-2-phenylindole (DAPI). Scale bars are indicated in (A), (D), and (H). Fluorescence intensity in (I) is normalized to the control group and expressed as fold change. Data are presented as mean ± standard deviation (SD) from three biologically independent pooled primary chondrocyte preparations (*n* = 3). Statistical significance was assessed using one-way analysis of variance (ANOVA) followed by Dunnett’s or Šídák’s multiple-comparison test, as appropriate. ns, not significant; ∗*p* < 0.05; ∗∗*p* < 0.01; ∗∗∗*p* < 0.001; ∗∗∗∗*p* < 0.0001.

Intracellular ROS was next measured using DCFH-DA fluorescence. IL-1β increased ROS accumulation relative to the control group, and 7 μM TCS alone also elevated ROS levels. The combined IL-1β + TCS treatment produced the strongest ROS signal, with a significantly higher ROS level than IL-1β treatment alone (Figure 8D). These results indicated that TCS increased oxidative stress in primary chondrocytes, particularly under inflammatory stimulation.

Real-time quantitative PCR (real-time qPCR) analysis was performed to evaluate whether TCS altered the expression of the prioritized candidate genes and the cartilage matrix-related marker *Col2a1*; the primer sequences are provided in Table 1. Both IL-1β and TCS increased *Cbr3* and *Mmp13* expression and decreased *Maoa*, *Avpr1a*, and *Col2a1* expression. *Cbr3* and *Mmp13* expression levels were higher in the combined IL-1β + TCS group than in the IL-1β-only group, while *Col2a1* expression was lowest in the combined-treatment group. Although *Maoa* and *Avpr1a* showed their lowest numerical expression levels in the combined-treatment group, these decreases were not significantly different from IL-1β treatment alone (Figure 8E).

**Table 1:** Real-time qPCR primer sequences

Genes	Forward (5′–3′)	Reverse (5′–3′)
*Col2a1*	AGGTGCTCAAGGTTCTCGTG	TTGGGGCCTTGTTCACCTTT
*Mmp13*	AAGCACCCCAAAACACCAGA	ACATGAGGTCTCGGGATGGA
*Avpr1a*	GCGTCTGCCTATATGCTGGT	ATGTTGCGCCAGATGTGGTA
*Maoa*	GGTTTTGGAAGCCCGGGATA	CCCATTCCTGAGCGTGTCTT
*Cbr3*	AACGTGCTGGTCAACAATGC	CGTAAGCTGAGTCTGGCCAA
*Gapdh*	GCTGAGAATGGGAAGCTGGT	AGTGATGGCATGGACTGTGG

CBR3 protein expression was further examined by western blotting and immunofluorescence. Western blot analysis showed a numerical, but non-significant, increase in CBR3 protein abundance after IL-1β treatment alone. In contrast, 7 μM TCS significantly increased CBR3 protein expression, and the highest CBR3 level was observed in the IL-1β + TCS group (Figures 8F and 8G). Consistently, CBR3 immunofluorescence intensity increased after IL-1β or TCS treatment and was strongest after combined treatment (Figures 8H and 8I).

Together, these cellular experiments supported the computational prioritization of CBR3. They further showed that TCS exposure increased ROS accumulation and induced OA-relevant transcriptional alterations in primary chondrocytes, with selected changes becoming more pronounced under IL-1β-stimulated conditions.

### Construction of a hypothesis-generating adverse outcome pathway (AOP)-like framework

Based on the integrated analyses, a hypothesis-generating AOP-like framework was developed to summarize the potential relationships among TCS-related candidate targets, OA-associated transcriptomic features, immune cell composition, and structural modeling results (Figure 9).

**Figure 9:**
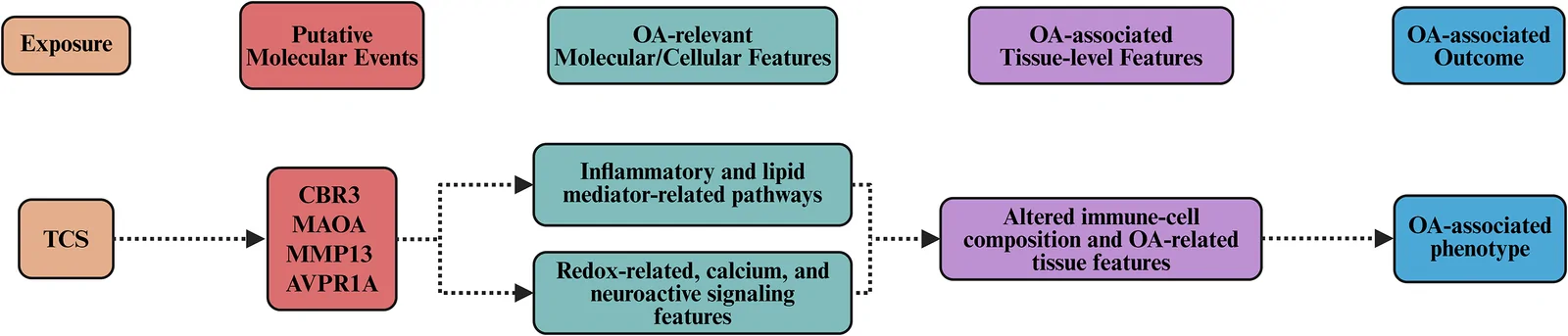
Hypothesis-generating AOP-like framework of TCS-related candidate genes and OA-associated features

In this exploratory framework, TCS is regarded as the chemical exposure of interest, with CBR3, MAOA, MMP13, and AVPR1A identified as putative molecular targets implicated in early molecular events. This identification is supported by target prediction, overlap with OA transcriptomic data, machine learning prioritization, and SHAP interpretation. Functional enrichment, single-cell analysis, immune infiltration analysis, molecular docking, and MD simulations further suggest associations between these candidate genes and OA-relevant inflammatory, redox-related, cellular, and structural characteristics.

Collectively, this AOP-like framework offers a conceptual and hypothesis-generating synthesis of the integrated findings. While it does not establish a validated causal adverse outcome pathway, it underscores the potential biological connections between TCS-related candidate targets and OA-associated molecular and cellular alterations, providing a foundation for future mechanistic validation.

## Discussion

This study aimed to investigate the convergence of TCS-related molecular perturbations with OA-relevant chondrocyte biology. Through the integration of network toxicology, bulk transcriptomics, machine learning prioritization, immune infiltration analysis, single-cell transcriptomic analysis, molecular interaction modeling, and primary chondrocyte experiments, CBR3, MAOA, MMP13, and AVPR1A were identified as candidate genes linking predicted TCS-related targets with OA-associated transcriptomic alterations. Key experimental observations revealed that low-dose TCS exposure did not result in uniform changes across all measured molecular endpoints. Instead, a selective pattern emerged, characterized by increased ROS accumulation, elevated expression of *Cbr3* and *Mmp13*, decreased expression of *Col2a1*, *Maoa*, and Avpr1a, and increased CBR3 protein levels and immunofluorescence intensity. This pattern supports a working model in which TCS-associated redox disturbances are linked to OA-like matrix remodeling in chondrocytes.

Redox stress appeared to be the primary point of convergence between computational predictions and cell-based findings. TCS exposure increased intracellular ROS under both basal and IL-1β-stimulated conditions, with coordinated increases in *Cbr3* and *Mmp13* and a reduction in *Col2a1* expression. These changes suggest that TCS-associated redox disturbances may facilitate a shift from matrix maintenance to catabolic remodeling in chondrocytes. Notably, ROS, Cbr3, and Mmp13 levels were higher in the combined-treatment group than in the IL-1β-only group, indicating that these changes were more pronounced under the combined treatment condition, although this pattern was not uniformly observed across all candidate genes. *Maoa* and *Avpr1a* did not exhibit significant changes beyond the effects of IL-1β, indicating that a nonspecific amplification of the entire inflammatory transcriptional program is unlikely. This selectivity is critical, as oxidative stress, mitochondrial dysfunction, altered metabolism, and redox-sensitive inflammatory signaling are recognized contributors to chondrocyte senescence, apoptosis, inflammatory amplification, and extracellular matrix imbalance in OA.^2^^,^^13^^,^^14^

The rationale for examining TCS concerning OA arises from two converging fields. First, human biomonitoring and environmental surveys indicate widespread TCS exposure through personal care products, disinfectants, and contaminated environmental media.^15^^,^^16^^,^^17^^,^^18^ Experimental toxicology has also linked TCS to ROS production across multiple biological systems.^14^ In immune, intestinal, and neural cell models, TCS has been reported to modify mitochondrial activity, alter glycolytic metabolism, and promote inflammatory activation, including inflammasome-related responses.^19^^,^^20^^,^^21^^,^^22^^,^^23^ Second, increasing attention has been directed toward environmental contributors to OA, with systematic reviews and epidemiological evidence implicating pollutants, such as particulate matter and phenolic exposures, as modifiers of OA susceptibility or symptom burden.^24^^,^^25^^,^^26^ Notably, an NHANES-based study on phenols did not find a significant correlation between urinary TCS and OA prevalence, although other phenolic compounds showed associations.^27^ Thus, the present study does not assert that TCS is an established population-level OA risk factor; rather, it explores whether TCS-related molecular events are biologically compatible with OA-relevant cellular processes.

This cellular interpretation aligns with the contemporary understanding of OA as a whole-joint disease rather than mere passive cartilage wear. OA encompasses involvement of the cartilage, synovium, subchondral bone, periarticular tissues, pain pathways, immune activation, metabolic stress, and vascular regulation.^2^^,^^28^^,^^29^^,^^30^ Recent molecular quantitative trait locus and synovial transcriptomic studies have provided genetic and regulatory insights into OA tissue heterogeneity.^31^^,^^32^ Single-cell studies have further revealed diverse chondrocyte, synovial, immune, fibroblast-like, and progenitor-like cell states in OA tissues, supporting the notion of coordinated changes across multiple joint-resident and immune cell populations.^9^^,^^33^^,^^34^ Within this framework, the candidate genes identified in this study are best viewed as nodes that may participate in, or mark, redox, matrix, and immune-stromal programs active in OA tissues. Notably, public OA datasets did not include individual TCS exposure measurements; thus, immune infiltration results should be interpreted as associations between candidate gene expression and inferred immune cell composition, rather than as evidence of TCS directly remodeling the OA immune microenvironment.

CBR3 emerges as the strongest candidate in this study, supported by both computational and experimental evidence. It was prioritized through the integrative workflow, increased at the mRNA level following TCS exposure, elevated at the protein level in western blot analyses, and displayed stronger immunofluorescence signals in treated chondrocytes. Its expression response paralleled ROS accumulation and was highest under the combined TCS and IL-1β treatment condition. This consistency across different layers distinguishes CBR3 from other candidates supported solely by transcriptomic evidence. Biologically, CBR3 is part of the short-chain dehydrogenase/reductase family and is linked to redox-responsive regulation; its transcription can be upregulated via the Nrf2 antioxidant response pathway and induced by pro-inflammatory stimuli.^35^^,^^36^ Thus, CBR3 may play a role in or serve as a marker of the cellular response to TCS-associated oxidative and inflammatory stress. Whether increased CBR3 expression is protective, maladaptive, or primarily a marker of stress adaptation remains to be determined and should be evaluated through targeted gain- and loss-of-function experiments.

MMP13 and COL2A1 provide the clearest matrix-level indicators related to the proposed redox-centered model. Matrix metalloproteinases are critical mediators of extracellular matrix proteolysis in arthritis, with MMP13 being a key collagenase involved in type II collagen degradation in OA cartilage.^37^^,^^38^ Preclinical studies utilizing *Mmp13* silencing, targeted delivery systems, and other regulatory strategies have further substantiated the functional significance of this enzyme in OA-related cartilage damage.^39^^,^^40^^,^^41^ In our experiments, TCS exposure was associated with increased *Mmp13* levels and reduced *Col2a1* expression, while the combined TCS and IL-1β group showed higher *Mmp13* and lower *Col2a1* expression than the IL-1β-only group. This pairing provides more informative insights than either marker alone: *Mmp13* upregulation indicates enhanced catabolic pressure, while *Col2a1* downregulation reflects diminished matrix-maintenance capacity. This pattern is consistent with IL-1β/NF-κB-associated chondrocyte catabolism,^42^^,^^43^ suggesting that TCS-associated redox stress may drive chondrocytes toward an OA-like matrix-remodeling state.

MAOA adds an additional layer to the model by linking the TCS response to mitochondrial and autophagy-related homeostasis. Located at the mitochondrial outer membrane, MAOA participates in monoamine metabolism, producing hydrogen peroxide and aldehydes that can influence mitochondrial stress and inflammatory signaling.^44^ In OA-related models, downregulation of MAOA has been observed in cartilage and IL-1β-treated chondrocytes, while restoration of MAOA has been shown to lessen chondrocyte loss and extracellular matrix degradation through autophagy-related mechanisms.^45^ The current data align directionally with these findings: IL-1β decreased *Maoa* expression, TCS exposure alone also led to decreased *Maoa* expression, and the combined treatment resulted in the lowest numerical level. However, the combined treatment did not significantly reduce *Maoa* below the level seen with IL-1β treatment alone. This suggests that while MAOA may indicate mitochondrial or autophagy-related vulnerabilities within the TCS-OA framework, it is unlikely to represent a primary contributor to the selected matrix-related changes observed under the combined treatment condition.

AVPR1A and the immune-context analyses should be considered auxiliary rather than central to the mechanistic framework. AVPR1A, a G-protein-coupled vasopressin receptor involved in calcium-dependent and neuroendocrine signaling,^46^^,^^47^ was retained in the integrative prioritization framework but exhibited weaker individual predictive performance, sparse expression at the single-cell level, and downregulation in both bulk OA transcriptomic data and treated primary chondrocytes. Additionally, *Avpr1a* expression was not significantly lower in the combined-treatment group compared to the IL-1β treatment group. These outcomes do not support AVPR1A as a primary mediator of the chondrocyte response observed in this study. Instead, AVPR1A may represent an exploratory link to neuroendocrine or calcium-signaling biology that could be relevant within the broader whole-joint environment, particularly in conjunction with immune-stromal associations found in OA datasets.

Molecular docking and MD simulations aided in candidate prioritization but should not be overextended. Computational interaction modeling helps identify structurally plausible protein-chemical pairs and organize downstream experimental priorities.^48^^,^^49^ In this study, these analyses connected predicted TCS-related targets with the transcriptomic and experimental candidate set. However, predicted binding compatibility does not confirm intracellular target engagement, target occupancy, or pathway activation. Thus, the modeling results should be viewed as a structural component within a broader AOP-like framework, not as definitive proof that TCS directly acts through CBR3, MAOA, MMP13, or AVPR1A in chondrocytes.^50^

Overall, the findings support a focused model in which TCS-associated redox disturbances are linked to selective chondrocyte stress and matrix-remodeling responses. In this model, redox disturbance serves as the central experimental phenotype, CBR3 is the most consistent redox-responsive candidate, MAOA reflects mitochondrial and autophagy-related vulnerabilities, and the MMP13-COL2A1 pair delineates the matrix-remodeling outcome axis. AVPR1A and immune-infiltration results extend the framework toward neuroendocrine and immune-stromal contexts but should remain exploratory. This structure preserves the main biological message of the study without overstating causality: TCS was not linked to a uniform amplification of all IL-1β-related responses; rather, the combined treatment condition exhibited greater changes in selected redox and matrix-catabolic features pertinent to OA biology. Future studies should investigate whether perturbations in CBR3 or MAOA influence ROS accumulation and whether targeting MMP13-related matrix responses can mitigate the chondrocyte changes associated with TCS exposure.

### Limitations of the study

This study has several limitations. First, the public OA datasets did not include individual-level TCS exposure information, which precludes direct assessment of exposure-response relationships. Additionally, while the transcriptomic analyses were primarily based on human samples, the experimental validation utilized primary rat chondrocytes. Second, the cell experiments were conducted using a single selected TCS concentration and a short exposure duration, limiting conclusions about dose-response relationships, chronic exposure effects, and reversibility. Third, protein-level validation was restricted to CBR3, and the functional roles of CBR3, MAOA, MMP13, and AVPR1A in the observed ROS and matrix-related changes were not directly investigated. Finally, although molecular docking and MD simulations supported predicted structural compatibility, they did not demonstrate direct intracellular target engagement. Future studies should incorporate dose-response and longer term exposure designs, target perturbation, direct binding assays, and *in vivo* validation to clarify causal directionality and biological relevance.

## Resource availability

### Lead contact

Further information and requests for resources should be directed to and will be fulfilled by the lead contact, Zhanghua Li (zhanghuali121@gmail.com).

### Materials availability

This study did not generate new unique reagents or materials.

### Data and code availability

• All data reported in this article will be shared by the lead contact upon request.
• This paper does not report original code.
• Any additional information required to reanalyze the data reported in this article is available from the lead contact upon request.

## Acknowledgments

This work was supported by the Scientific Research Project of Health Commission of Hubei Province (no. WJ2023ZH0025), Natural Science Foundation of Hubei Province (no. 2025AFD655), 14th Five-Year-Plan Advantageous and Characteristic Disciplines (Groups) of Colleges and Universities in Hubei Province, and Wuhan Natural Science Foundation Clinical Research Key Project of Municipal Medical Institutions (no. 2026020301040138).

## Author contributions

X.T., writing – original draft, conceptualization, methodology, and software; H.X., writing – original draft, writing – review and editing, validation, and funding acquisition; X.L., validation; Y.Y., conceptualization, validation, supervision, and funding acquisition; Z.L., writing – review and editing, conceptualization, validation, supervision, and funding acquisition.

## Declaration of interests

The authors declare no competing interests.

## STAR★Methods

### Key resources table

**Table:** 

REAGENT or RESOURCE	SOURCE	IDENTIFIER
**Antibodies**		

Rabbit anti-CBR3 primary antibody	Proteintech	Cat#15619-1-AP; RRID: AB_2070862
Anti-β-actin primary antibody	Servicebio	Cat#GB15003-100; RRID: AB_3083699
HRP-conjugated goat anti-rabbit IgG secondary antibody	Servicebio	Cat#GB23303; RRID: AB_2811189
Cy3-conjugated goat anti-rabbit IgG secondary antibody	Servicebio	Cat#GB21303; RRID: AB_2861435

**Chemicals, peptides, and recombinant proteins**		

Triclosan (TCS)	Meilunbio	Cat#MB1842-S
Interleukin-1β (IL-1β)	MedChemExpress	Cat#HY-P7097A
0.25% Trypsin-EDTA	Biosharp	Cat#BL501A
Type II collagenase	MedChemExpress	Cat#HY-E70005B
Toluidine blue staining solution	Servicebio	Cat#G1032-100 ML
RIPA lysis buffer	Servicebio	Cat#G2002-30 ML
Protease inhibitor(s)	Servicebio	Cat#G2007-1 ML
Ready-to-use DAPI staining solution	Servicebio	Cat#G1012-10 ML

**Critical commercial assays**		

Cell Counting Kit-8 (CCK-8) assay	Servicebio	Cat#G4103-1 ML
DCFH-DA fluorescent probe kit	Meilunbio	Cat#MA0219-1
RNA extraction kit	Omega	Cat#R6834-01
Reverse-transcription kit	TOYOBO	Cat#FSQ-101
SYBR Green qPCR master mix	CWBIO	Cat#CW3390M
Bicinchoninic acid (BCA) protein assay kit	Servicebio	Cat#G2026-200T

**Deposited data**		

OA bulk transcriptomic dataset GSE55235	NCBI Gene Expression Omnibus (GEO)	GEO: GSE55235
OA bulk transcriptomic dataset GSE55457	NCBI Gene Expression Omnibus (GEO)	GEO: GSE55457
OA bulk transcriptomic dataset GSE82107	NCBI Gene Expression Omnibus (GEO)	GEO: GSE82107
OA bulk transcriptomic dataset GSE57218	NCBI Gene Expression Omnibus (GEO)	GEO: GSE57218
OA bulk transcriptomic dataset GSE46750	NCBI Gene Expression Omnibus (GEO)	GEO: GSE46750
OA bulk transcriptomic dataset GSE206848	NCBI Gene Expression Omnibus (GEO)	GEO: GSE206848
OA single-cell RNA-seq dataset GSE169454	NCBI Gene Expression Omnibus (GEO)	GEO: GSE169454
AVPR1A protein structure	RCSB Protein DataBank (PDB)	PDB: 9UWL
CBR3 protein structure	RCSB Protein DataBank (PDB)	PDB: 2HRB
MAOA protein structure	RCSB Protein DataBank (PDB)	PDB: 2Z5Y
MMP13 protein structure	RCSB Protein DataBank (PDB)	PDB: 3TVC

**Software and algorithms**		

R version 4.6.0	https://www.r-project.org/	4.6.0
PyMOL version 2.6.0	https://pymol.org/	2.6.0
AutoDockTools version 1.5.6	https://ccsb.scripps.edu/autodocksuite/adt/	1.5.6
AutoDock Vina version 1.1.2	https://vina.scripps.edu/	1.1.2
GROMACS 2020	Abraham et al.^51^	N/A
AMBER99SB-ILDN force field	Lindorff-Larsen et al.^52^	N/A
General Amber Force Field (GAFF)	Wang et al.^53^	N/A
LINCS algorithm	Hess et al.^54^	N/A
ImageJ version 1.54g	https://imagej.net	1.54g
GraphPad Prism version 10	https://www.graphpad.com	10

**Other**		

PubChem chemical data resource	PubChem	https://pubchem.ncbi.nlm.nih.gov/
SwissTargetPrediction target prediction resource	SwissTargetPrediction	https://swisstargetprediction.ch/
STITCH chemical-protein interaction resource	STITCH	http://stitch.embl.de/
ChEMBL bioactivity database	ChEMBL	https://www.ebi.ac.uk/chembl/
Similarity Ensemble Approach (SEA) target prediction resource	Similarity Ensemble Approach	https://sea.bkslab.org/
SpectraMax CMax Plus absorbance reader	Molecular Devices	N/A
Leica DMI1 LED inverted microscope	Leica	N/A
LG-FS80 fluorescence slide scanner	202510-FS80-0005#	V1.0.4.6
Enhanced chemiluminescence detection system	Bio-Rad	N/A

### Experimental model and study participant details

Publicly available human osteoarthritis (OA) transcriptomic datasets were obtained from the NCBI Gene Expression Omnibus (GEO) database. GSE55235, GSE55457, and GSE82107 were integrated and used as the training cohort, whereas GSE57218, GSE46750, and GSE206848 were used as independent external evaluation cohorts. GSE55235 included 10 control and 20 OA samples; GSE55457 included 10 control and 23 OA samples; GSE82107 included 7 control and 10 OA samples; GSE57218 included 7 control and 33 OA samples; GSE46750 included 12 control and 12 OA samples; and GSE206848 included 7 control and 9 OA samples. The OA-related single-cell RNA sequencing dataset GSE169454, comprising seven samples, was used for single-cell transcriptomic analysis. No new human participants or human biological samples were prospectively recruited or collected in this study.

Primary rat chondrocytes were isolated from 4-week-old male Sprague-Dawley rats as described below under “Primary chondrocyte culture and cell-based assays.” Only male rats were used; therefore, potential sex-related differences were not evaluated. All animal procedures were approved by the Experimental Animal Ethics Committee of Wuhan Third Hospital (No. 25-002) and conducted in accordance with relevant institutional guidelines and regulations for animal care and use.

### Method details

#### Identification of OA-related genes

For the training cohort, probe IDs were converted to official gene symbols according to the corresponding platform annotation files. When multiple probes mapped to the same gene symbol, the average expression value was used. To reduce inter-dataset technical variation, the three training datasets were merged, and batch effects were corrected using the ComBat algorithm implemented in the sva R package. PCA was performed before and after batch correction to evaluate the effectiveness of batch-effect removal. The external evaluation cohorts were processed separately and were not included in the batch correction of the training cohort.

Differential expression analysis between OA and normal samples was performed using the limma R package based on the batch-corrected training expression matrix. Differentially expressed genes (DEGs) were identified according to both statistical significance and effect-size criteria: Benjamini-Hochberg FDR <0.05 and |log2FC| > 0.585. The threshold of |log2FC| > 0.585 corresponds to a minimum 1.5-fold expression difference between OA and normal samples, as log2(1.5) = 0.585. Volcano plots and heatmaps were generated to visualize the distribution and expression patterns of DEGs.

In parallel, weighted gene co-expression network analysis (WGCNA) was performed using the same batch-corrected training expression matrix to identify OA-associated gene modules. Prior to network construction, hierarchical clustering was used to detect potential outlier samples. An unsigned co-expression network was then constructed. The soft-thresholding power was selected according to the scale-free topology criterion. A power of β = 7 was chosen because it exceeded the predefined threshold of R^2^ > 0.85, with an observed scale-free topology fit index of approximately 0.93, while maintaining acceptable mean connectivity.

Gene modules were identified by hierarchical clustering based on the topological overlap matrix (TOM) and dynamic tree cutting. To improve the sensitivity for detecting smaller biologically relevant co-expression modules while avoiding excessive module fragmentation, the minimum module size was set to 100 genes. Modules with highly similar eigengenes were merged using a cut height of 0.25.

The association between each module and OA status was evaluated by calculating the correlation between module eigengenes and disease phenotype. Modules significantly associated with OA were defined as OA-related modules, and genes within these modules were extracted. The DEG list and genes from OA-associated WGCNA modules were then combined as a union to generate a comprehensive OA-related gene set for downstream integration with TCS-related targets.

#### Acquisition of TCS chemical information and predicted target genes

The chemical structure and molecular properties of triclosan (TCS) were retrieved from the PubChem database. The standardized two-dimensional structure and canonical SMILES notation were obtained as follows: C1 = CC(=C(C=C1Cl)O)OC2 = C(C=C(C=C2)Cl)Cl.

Putative human target genes of TCS were collected from SwissTargetPrediction, STITCH, ChEMBL, and the Similarity Ensemble Approach (SEA) database. Target lists obtained from these databases were integrated, and duplicate entries were removed to generate a non-redundant set of predicted TCS-related target genes for subsequent intersection analysis.

#### Identification of shared TCS- and OA-related candidate genes and functional enrichment analysis

To identify candidate genes shared between predicted TCS-related targets and OA-associated transcriptomic alterations, the OA-related gene set was intersected with the predicted TCS-related target gene set. Genes present in both datasets were defined as shared TCS- and OA-related candidate genes and were used for subsequent functional enrichment, machine-learning prioritization, and validation analyses.

Functional enrichment analysis was performed to explore the biological processes and pathways associated with the shared TCS- and OA-related candidate genes. GO enrichment analysis was conducted for biological process (BP), cellular component (CC), and molecular function (MF) categories. KEGG pathway enrichment analysis was also performed. Enrichment analyses were conducted using R-based bioinformatics tools, and the Benjamini-Hochberg FDR method was applied to correct for multiple testing. Terms or pathways with FDR-adjusted *p* values <0.05 were considered significantly enriched. The enrichment results were visualized using bar plots and bubble plots.

#### Machine-learning-based prioritization and SHAP interpretation of shared TCS- and OA-related candidate genes

To prioritize robust candidate genes among the shared TCS- and OA-related candidate gene set, a machine-learning framework was constructed based on their expression profiles in the training cohort. A total of 30 shared TCS- and OA-related candidate genes were used as the initial input features. Twelve commonly used machine-learning algorithms were incorporated, including least absolute shrinkage and selection operator (LASSO) regression, ridge regression, stepwise generalized linear model (Stepglm), extreme gradient boosting (XGBoost), random forest (RF), elastic net (Enet), partial least-squares regression for generalized linear models (plsRglm), generalized boosted regression modeling (GBM), naive Bayes, linear discriminant analysis (LDA), generalized linear model boosting (glmBoost), and support vector machine (SVM).

Different feature-selection and classification algorithms were combined to generate 113 predefined machine-learning pipelines. Model training and hyperparameter tuning were performed in the training cohort using repeated stratified 5-fold cross-validation, with 5-folds repeated 20 times, generating 100 resampling iterations. During each resampling procedure, feature selection and model fitting were conducted within the training partitions to reduce the risk of information leakage. Model performance was primarily evaluated using the area under the receiver operating characteristic curve (AUC).

The three independent external evaluation cohorts, including GSE57218, GSE46750, and GSE206848, were not used for feature selection, model fitting, or hyperparameter tuning within the training cohort. Candidate pipelines were first internally assessed using repeated stratified 5-fold cross-validation in the training cohort. Each pipeline was subsequently fitted using the full training cohort and evaluated in the three independent external cohorts to assess cross-cohort performance. For final pipeline prioritization, the mean AUC across the training cohort and the three external evaluation cohorts was used as the summary performance metric. Thus, the external cohorts contributed to cross-cohort model evaluation and final pipeline prioritization, but not to model fitting or hyperparameter optimization.

The 113 machine-learning pipelines were predefined predictive modeling strategies and were not treated as independent statistical hypotheses. Therefore, no formal pairwise hypothesis testing was performed among pipelines, and multiple-comparison-adjusted *p* values were not applied to model ranking. Instead, model selection was based on predictive performance metrics, with AUC used as the primary criterion. The pipeline with the highest mean AUC across the training cohort and the three independent external evaluation cohorts was selected as the final predictive model. Genes retained by the final model were defined as prioritized candidate genes for subsequent analyses.

To improve model interpretability and assess the contribution of individual genes to model prediction, SHAP analysis was performed for the final model. SHAP values were calculated to quantify the contribution of each gene to the predicted OA classification. To reduce dependence on a single fitted model, SHAP analysis was further repeated across 100 stratified resampling iterations. Mean absolute SHAP values and rank stability were summarized to evaluate the robustness of gene importance. Genes showing consistent predictive contributions and stable rankings across resampling analyses were prioritized for downstream single-cell analysis, immune infiltration analysis, molecular docking, molecular dynamics simulation, and experimental validation.

#### Single-cell RNA sequencing analysis

To investigate the cellular distribution of the prioritized candidate genes at single-cell resolution, publicly available OA-related single-cell RNA sequencing (scRNA-seq) data were obtained from the GEO database under accession number GSE169454.^55^ Gene expression matrices were processed using the Seurat R package.

Quality control was performed based on the number of detected genes, UMI count, mitochondrial gene percentage, and hemoglobin-related gene percentage. Cells were retained for downstream analysis if they met the following criteria: 300 < nFeature_RNA <5,000, nCount_RNA >1,000, mitochondrial gene percentage <10%, hemoglobin-related gene percentage <3%, and nCount_RNA below the 97th percentile within each sample. After quality control, individual samples were merged for integrated analysis.

Gene expression values were normalized using the NormalizeData function. The top 3,000 highly variable genes were identified using the variance-stabilizing transformation method, followed by data scaling and principal component analysis (PCA) with 50 principal components. To reduce sample-specific batch effects, Harmony integration was performed using sample identity as the batch variable. Cell clustering was performed using the first 20 Harmony-corrected dimensions with a shared nearest-neighbor graph at a resolution of 1.5, and UMAP was used for two-dimensional visualization.

Cluster-enriched marker genes were first identified using the FindAllMarkers function by comparing each cluster with all remaining cells. Cell-subpopulation annotation was then performed by integrating cluster-specific marker genes derived from the present dataset with previously reported OA chondrocyte subpopulation signatures. Representative marker genes supporting cell-subpopulation annotation were selected for heatmap visualization and supplementary summary.

The expression patterns of prioritized candidate genes were visualized across annotated cell subpopulations using UMAP-based feature plots. The proportion of cells with detectable expression of each prioritized candidate gene was also calculated within each annotated subpopulation to characterize their cell-subpopulation-specific expression distribution.

#### Immune infiltration and gene–immune cell correlation analysis

To characterize immune-cell composition associated with OA, immune infiltration analysis was performed using the CIBERSORT deconvolution algorithm. The batch-corrected bulk transcriptomic matrix from the training cohort was used as input, and the relative abundance of 22 immune cell subtypes was estimated based on the LM22 leukocyte signature matrix.

Differences in immune-cell abundance between OA and normal samples were assessed using Wilcoxon rank-sum tests, followed by Benjamini-Hochberg FDR correction. Spearman correlation analysis was performed to evaluate correlations among immune cell subtypes and the relationships between the expression levels of prioritized candidate genes and estimated immune-cell abundance. Benjamini-Hochberg FDR correction was applied to account for multiple correlation testing. Mantel tests were additionally used to summarize the associations between prioritized candidate genes and the overall immune-cell composition. Immune infiltration patterns and gene–immune cell associations were visualized using stacked bar plots, boxplots, immune-cell correlation plots, and combined gene–immune cell correlation network plots.

#### Molecular docking analysis

Molecular docking simulations were performed to evaluate the predicted binding interactions between TCS and the prioritized candidate proteins. The molecular structure of TCS was obtained from PubChem and saved in SDF format. Three-dimensional structures of the candidate proteins were retrieved from the PDB, including AVPR1A (9UWL), CBR3 (2HRB), MAOA (2Z5Y), and MMP13 (3TVC).

Protein structures were preprocessed using PyMOL by removing water molecules and co-crystallized ligands. Docking grid boxes were generated using the GetBox plugin to cover the putative ligand-binding regions. Protein and ligand structures were converted into PDBQT format using AutoDockTools version 1.5.6, and docking simulations were performed using AutoDock Vina version 1.1.2. For each protein–ligand pair, the docking pose with the lowest predicted binding energy was selected for further visualization and analysis. Docking conformations were visualized using PyMOL version 2.6.0. The predicted binding energies were used to provide an approximate comparison of the relative binding affinities between TCS and the candidate proteins.

#### Molecular dynamics simulation

MD simulations were performed to evaluate the conformational stability of the docked TCS–protein complexes using GROMACS 2020.^51^ The optimal docking conformations were used as the initial structures. The AMBER99SB-ILDN force field was applied for proteins,^52^ and ligand parameters for TCS were generated using the General Amber Force Field (GAFF) with AM1-BCC charges through ACPYPE/AmberTools.^53^ Each complex was solvated in a cubic TIP3P water box with a minimum distance of 1.0 nm from the box boundary, neutralized with Na^+^ and Cl^−^ ions, and adjusted to an ionic concentration of approximately 0.15 M.

Energy minimization was performed using the steepest descent algorithm until the maximum force was below 100 kJ mol^−1^ nm^−1^. The systems were then equilibrated first under the NVT ensemble at 310 K and subsequently under the NPT ensemble at 310 K and 1 bar before production MD simulations. Production MD simulations were conducted for 100 ns with a 2-fs time step. Bond constraints were applied using the LINCS algorithm,^54^ short-range non-bonded interactions were treated with a cutoff of 1.0 nm, and long-range electrostatic interactions were calculated using the particle mesh Ewald method as implemented in GROMACS.^51^

Trajectory analyses were performed using built-in GROMACS tools after removing periodic boundary effects and fitting the trajectories to the protein backbone of the initial structures. RMSD, RMSF, Rg, SASA, and protein–ligand hydrogen bonds were calculated to assess structural stability and interaction dynamics. FEL analysis was performed using gmx sham at 310 K, with protein backbone RMSD and Rg used as reaction coordinates, to visualize the conformational energy distribution of each complex.

#### Primary chondrocyte culture and cell-based assays

All animal procedures were approved by the Experimental Animal Ethics Committee of Wuhan Third Hospital (No. 25-002) and conducted in accordance with relevant institutional guidelines and regulations for animal care and use. Rats were euthanized by CO_2_ inhalation in accordance with the approved institutional protocol before articular cartilage collection. Primary rat chondrocytes were isolated from the knee articular cartilage of 4-week-old male Sprague-Dawley rats. A total of six rats were used for primary chondrocyte isolation. For each independent isolation, articular cartilage obtained from two rats was pooled to generate one primary chondrocyte preparation. Three independent isolations were performed, resulting in three biologically independent pooled primary chondrocyte preparations (*n* = 3). Articular cartilage tissues were aseptically harvested, washed with sterile phosphate-buffered saline (PBS), and cut into small fragments. The fragments were initially digested with 0.25% trypsin-EDTA (Biosharp, China) at 37 °C for 20 min, followed by digestion with 0.2% type II collagenase (MedChemExpress, USA) at 37 °C for 6 h. After digestion, the cell suspension was filtered through a 40-μm cell strainer and centrifuged at 1,200 rpm for 8 min. The collected cells were resuspended in DMEM/F-12 medium (Meilunbio, China) supplemented with 10% fetal bovine serum (FBS; Meilunbio, China) and 1% penicillin-streptomycin (Beyotime, China), and cultured at 37 °C in a humidified atmosphere containing 5% CO_2_. The culture medium was refreshed every 2–3 days. Chondrocytes between passages 1 and 3 were used for subsequent experiments to minimize phenotypic drift.

Toluidine blue staining was performed to evaluate the chondrocytic phenotype of the isolated primary cells. Chondrocytes were seeded in 24-well plates at a density of approximately 6.0 × 10^4^ cells per well and cultured until appropriate confluence was reached. The cells were washed with PBS and fixed in 4% paraformaldehyde (Servicebio, China) for 30 min at room temperature. After fixation, the cells were washed with PBS and stained with toluidine blue staining solution (Servicebio, China) for 30 min. Excess dye was removed by washing with distilled water, and the stained cells were observed and photographed under an inverted microscope (Leica DMI1 LED, Germany). Toluidine blue staining was utilized to assess glycosaminoglycan-rich extracellular matrix production, a characteristic feature of chondrocytes.

TCS (Meilunbio, China) was initially dissolved in dimethyl sulfoxide (DMSO; Meilunbio, China) to create a 50 mM stock solution, stored at −20 °C and protected from light. Prior to treatment, the stock solution was serially diluted and subsequently added to DMEM/F-12 medium containing 2% FBS and 1% penicillin-streptomycin to achieve the desired final treatment concentrations. The final concentration of DMSO was consistently maintained at 0.1% across all groups, including the control group.

Cell viability was assessed using a CCK-8 assay (Servicebio, China). Primary rat chondrocytes were seeded into 96-well plates at a density of 5 × 10^3^ cells/well and allowed to adhere overnight. The culture medium was then replaced with low-serum medium containing various concentrations of TCS. For the initial broad-range screening, cells were treated with 0, 5, 10, or 20 μM TCS. For further refined concentration screening, cells received treatments of 0, 5, 6, 7, 8, 9, or 10 μM TCS. The 0 μM group, which contained 0.1% DMSO, served as the control. After 30 h of treatment, 10 μL of CCK-8 solution was added to each well, followed by incubation at 37 °C for 2 h. Absorbance was then measured at 450 nm using a SpectraMax CMax Plus absorbance reader (Molecular Devices, USA), with wells without cells serving as blanks. Cell viability was calculated following blank subtraction and normalized to the control group. For each biological replicate, each condition was assessed using at least three technical replicate wells. The assay was performed using three biologically independent pooled primary chondrocyte preparations (*n* = 3). Based on the refined concentration-screening results, 7 μM TCS was chosen for subsequent cell-based assays.

#### Intracellular reactive oxygen species detection

Intracellular ROS levels were assessed using a DCFH-DA fluorescent probe kit (Meilunbio, China). Primary rat chondrocytes were seeded in 24-well plates and divided into four groups: Control, IL-1β, TCS, and IL-1β + TCS. Cells in the Control group were treated with 0.1% DMSO for 30 h. Cells in the IL-1β group were treated with 0.1% DMSO for 6 h, followed by stimulation with 10 ng/mL IL-1β in the continuous presence of 0.1% DMSO for an additional 24 h. Cells in the TCS group were treated with 7 μM TCS for 30 h. Cells in the IL-1β + TCS group were pretreated with 7 μM TCS for 6 h, followed by stimulation with 10 ng/mL IL-1β in the presence of 7 μM TCS for another 24 h.

After treatment, the culture medium was removed, and the cells were incubated with 10 μM DCFH-DA diluted in serum-free medium at 37 °C for 30 min in the dark. Cells were then washed three times with PBS to eliminate the extracellular probe, and fresh PBS was added before imaging. Intracellular DCF fluorescence images were captured using a fluorescence microscope under uniform exposure and acquisition settings across all groups. Each group was analyzed through three replicate wells for each biological replicate, with three randomly selected fields captured from each well. Fluorescence intensities of the three fields were averaged to obtain a single value for each well, and these values were further averaged across replicate wells for each biological replicate. The experiment was performed using three biologically independent pooled primary chondrocyte preparations (*n* = 3). Mean fluorescence intensity was quantified using ImageJ software and normalized to that of the Control group.

#### RT-qPCR

Total RNA was extracted from primary rat chondrocytes following the indicated treatments using an RNA extraction kit, in accordance with the manufacturer’s instructions. RNA concentration and purity were assessed using a spectrophotometer, and equal amounts of total RNA were reverse-transcribed into complementary DNA (cDNA) with a reverse-transcription kit.

Quantitative PCR was conducted using an SYBR Green-based qPCR master mix on a real-time PCR detection system, adhering to the manufacturer’s guidelines. Melting-curve analysis was performed post-amplification to confirm the specificity of the products. Relative expression levels of *Mmp13*, *Col2a1*, *Cbr3*, *Maoa*, and *Avpr1a* were measured, with *Gapdh* serving as the internal reference gene. Each sample was analyzed in technical triplicate. The experiment was performed using three biologically independent pooled primary chondrocyte preparations (*n* = 3). Relative gene expression was calculated using the 2^−ΔΔCq^ method and normalized to the Control group. The primer sequences are detailed in Table 1.

#### Western blot analysis

Total proteins were extracted from primary rat chondrocytes following the indicated treatments using RIPA lysis buffer (Servicebio, China) supplemented with protease inhibitors (Servicebio, China). Protein concentrations were measured using a bicinchoninic acid (BCA) assay kit (Servicebio, China). Equal amounts of protein were separated by 10% SDS-PAGE and transferred onto PVDF membranes. After blocking with 5% non-fat milk prepared in TBST at room temperature for 1 h, the membranes were incubated overnight at 4 °C with primary antibodies against CBR3 (Proteintech, China) and β-actin (Servicebio, China).

Following three washes with TBST, the membranes were incubated with an HRP-conjugated goat anti-rabbit IgG secondary antibody (Servicebio, China) at room temperature for 1 h. Protein bands were visualized using an enhanced chemiluminescence detection system (Bio-Rad, USA) and subjected to densitometric analysis using ImageJ software. Relative CBR3 protein expression was calculated as the ratio of CBR3 to β-actin band intensities and normalized to the Control group. The experiment was performed using three biologically independent pooled primary chondrocyte preparations (*n* = 3).

#### Immunofluorescence staining

Primary rat chondrocytes were seeded on sterile coverslips and divided into four groups: Control, IL-1β, TCS, and IL-1β + TCS. Cells in the Control group were treated with 0.1% DMSO for 30 h. Cells in the IL-1β group received 0.1% DMSO for 6 h, followed by stimulation with 10 ng/mL IL-1β in the continued presence of 0.1% DMSO for an additional 24 h. The TCS group was treated with 7 μM TCS for 30 h, while the IL-1β + TCS group was pretreated with 7 μM TCS for 6 h, followed by stimulation with 10 ng/mL IL-1β in the presence of 7 μM TCS for another 24 h.

After treatment, the cells were washed with PBS and fixed in 4% paraformaldehyde (Servicebio, China) for 15 min at room temperature. Subsequently, cells were permeabilized with 0.1% Triton X-100 (Servicebio, China) for 10 min and blocked with 3% bovine serum albumin for 1 h at room temperature. Cells were incubated overnight at 4 °C with a rabbit anti-CBR3 primary antibody (Proteintech, China). Following three washes with PBS, the cells were incubated with a Cy3-conjugated goat anti-rabbit IgG secondary antibody (Servicebio, China) for 1 h at room temperature in the dark.

Cell nuclei were counterstained with a ready-to-use DAPI staining solution (Servicebio, China) for 10 min at room temperature in the dark, followed by three PBS washes. Fluorescence images were captured using an LG-FS80 fluorescence slide scanner equipped with a 20× Plan-Apochromat objective under identical exposure and acquisition settings for all groups. For each biological replicate, three randomly selected fields were captured for each group, and the fluorescence intensities of these fields were averaged to yield one value per biological replicate. CBR3 fluorescence intensity was quantified using ImageJ software, normalized to the Control group, and expressed as fold change. The experiment was performed using three biologically independent pooled primary chondrocyte preparations (*n* = 3).

#### Construction of a hypothesis-generating AOP-like framework

An AOP-like framework was developed to summarize the putative biological relationships indicated by the integrated analyses. This framework combines shared TCS- and OA-related candidate genes, enriched biological processes and pathways, prioritized candidate genes, immune infiltration patterns, single-cell expression distributions, molecular docking results, molecular dynamics simulations, and findings from experimental validations. These components were organized to elucidate potential molecular initiating events, intermediate biological events, and OA-related outcomes.

The proposed framework is interpreted as exploratory rather than a formally validated adverse outcome pathway. Key-event relationships are considered inferential and serve to guide biological interpretation and future experimental validation.

### Quantification and statistical analysis

#### Statistical analysis for cell experiments

Cell-based experiments were performed using three biologically independent pooled primary chondrocyte preparations unless otherwise specified. Each preparation was generated from articular cartilage pooled from two separate rats and was considered one biological replicate (*n* = 3; six rats in total). Data are presented as mean ± SD. Statistical analyses were conducted using GraphPad Prism version 10. For CCK-8 concentration-screening assays, comparisons with the Control group were performed using one-way ANOVA followed by Dunnett’s multiple-comparison test. For ROS, RT-qPCR, western blotting, and immunofluorescence analyses, the four treatment conditions were analyzed as predefined experimental groups using one-way ANOVA followed by Šídák-adjusted multiple comparisons. This approach was used because the primary objective was to evaluate prespecified between-group differences rather than to estimate the main effects of TCS and IL-1β or formally test a TCS × IL-1β interaction. Accordingly, no statistical interaction or synergistic effect was inferred from these analyses. A *p* value <0.05 was considered statistically significant. ns, not significant; ∗*p* < 0.05; ∗∗*p* < 0.01; ∗∗∗*p* < 0.001; ∗∗∗∗*p* < 0.0001.

## Supplemental information

Data S1. Full gel images
