# Supplementary material for: Potential molecular links between triclosan exposure and osteoarthritis: An integrative systems toxicology study
Source: iScience. 2026 Oct 13;29(11):117868. doi: 10.1016/j.isci.2026.117868 (PMC13682359; doi:10.1016/j.isci.2026.117868)

iScience, Volume *29*

Supplemental information

Potential molecular links between triclosan exposure and osteoarthritis: An integrative systems toxicology study

Xijie Tang, Haijia Xu, Xiangzhong Liu, Yi Yang, and Zhanghua Li

Data S1 Full gel images

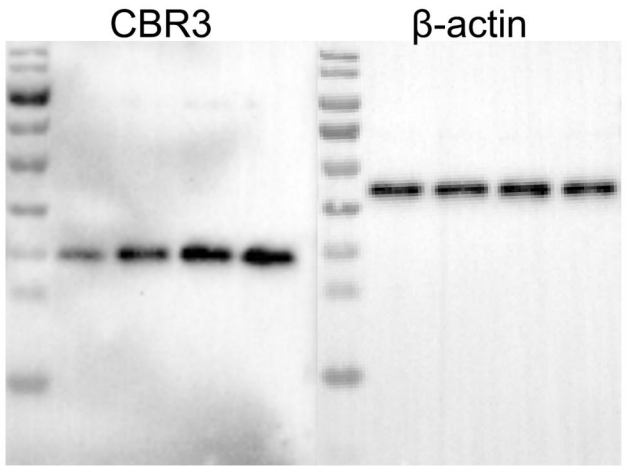

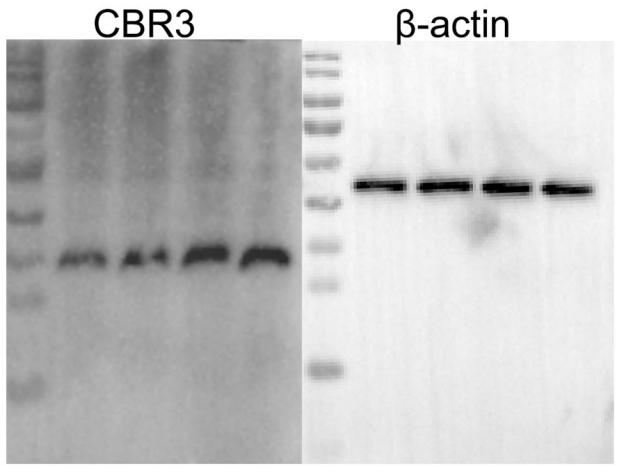

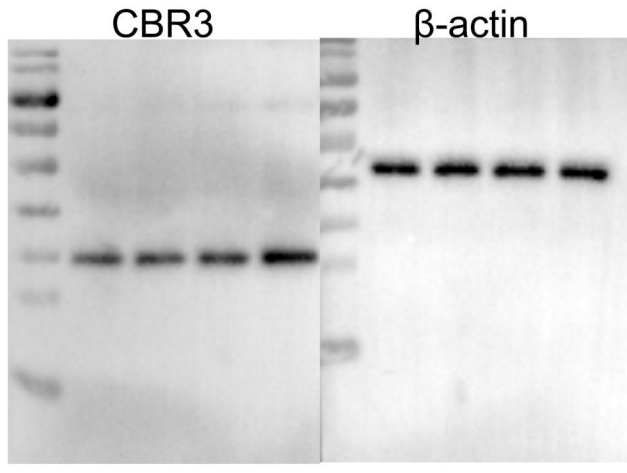

Supplement: Supplementary file 1 [file mmc1.pdf]
